# Computational modeling of DLBCL predicts response to BH3-mimetics

**DOI:** 10.1038/s41540-023-00286-5

**Published:** 2023-06-06

**Authors:** Ielyaas Cloete, Victoria M. Smith, Ross A. Jackson, Andrea Pepper, Chris Pepper, Meike Vogler, Martin J. S. Dyer, Simon Mitchell

**Affiliations:** 1 Brighton and Sussex Medical School, University of Sussex, Brighton, UK; 2 Department of Molecular and Cell Biology, University of Leicester, Leicester, UK; 3 The Ernest and Helen Scott Haematological Research Institute, Leicester Cancer Research center, University of Leicester, Leicester, UK; 4 Institute for Experimental Cancer Research in Pediatrics, Goethe-University, Frankfurt, Germany

**Keywords:** Computer modelling, Cell biology, Cancer

## Abstract

In healthy cells, pro- and anti-apoptotic BCL2 family and BH3-only proteins are expressed in a delicate equilibrium. In contrast, this homeostasis is frequently perturbed in cancer cells due to the overexpression of anti-apoptotic BCL2 family proteins. Variability in the expression and sequestration of these proteins in Diffuse Large B cell Lymphoma (DLBCL) likely contributes to variability in response to BH3-mimetics. Successful deployment of BH3-mimetics in DLBCL requires reliable predictions of which lymphoma cells will respond. Here we show that a computational systems biology approach enables accurate prediction of the sensitivity of DLBCL cells to BH3-mimetics. We found that fractional killing of DLBCL, can be explained by cell-to-cell variability in the molecular abundances of signaling proteins. Importantly, by combining protein interaction data with a knowledge of genetic lesions in DLBCL cells, our in silico models accurately predict in vitro response to BH3-mimetics. Furthermore, through virtual DLBCL cells we predict synergistic combinations of BH3-mimetics, which we then experimentally validated. These results show that computational systems biology models of apoptotic signaling, when constrained by experimental data, can facilitate the rational assignment of efficacious targeted inhibitors in B cell malignancies, paving the way for development of more personalized approaches to treatment.

## Introduction

Apoptosis is a crucial component for the development of multicellular organisms and the functioning of the immune system. The BCL2 family of proteins are the principal regulators of mitochondrial-dependent apoptosis. This family of proteins consists of more than twenty-five members, and is further categorized into three groups based on their protein structure and function (Supplementary Fig. 1A and B): pro-apoptotic BCL2 proteins (BAX and BAK), anti-apoptotic BCL2 proteins (BCL2, BCL-xL, MCL1, etc.) and BCL2 homology domain 3 (BH3)-only proteins (BID, BIM, PUMA, NOXA, etc.)^1^. Prior to initiation of apoptosis, anti-apoptotic BCL2 proteins bind to BAX/BAK proteins at the mitochondrial outer membrane (MOM), impeding BAX/BAK oligomerization, which prevents mitochondrial outer membrane permeabilization (MOMP). Initiation of apoptosis leads to activation of BH3-only proteins, which either activate BAX/BAK directly through complex formation between BH3-only proteins and BAX/BAK or activate BAX/BAK indirectly by sequestering anti-apoptotic BCL2 proteins leading to the release of BAX/BAK from complexes containing the anti-apoptotic BCL2 proteins. BAX/BAK activation results in MOMP and subsequent apoptotic cell death^2^.

Avoidance of apoptosis is a hallmark of cancer, which in B cell lymphoma is often achieved through the upregulation of anti-apoptotic BCL2 proteins due to chromosomal translocation, gene amplification or constitutive activation of transcription factors that upregulate BCL2 family proteins such as nuclear factor kappa B (NF-κB)^3–5^. BCL2 dysregulation is commonly linked to chemoresistance and poor prognosis, and therefore represents a pathologically important biomarker and an attractive therapeutic target in B cell lymphoma^6,7^.

ABT-199 (venetoclax), a BCL2 specific inhibitor, was first approved for treatment of chronic lymphocytic leukemia (CLL) and acute myeloid leukemia^8–10^, and has shown significant clinical activity in CLL regardless of genotype^11^. However, in Diffuse Large B cell Lymphoma (DLBCL), responses to ABT-199 are less impressive, despite BCL2 overexpression in about 40% of cases^8^. In a similar vein, BCL-xL is highly expressed in about 95% of DLBCL patient samples but only a proportion of DLBCL cell lines respond to BCL-xL inhibition^12,13^. Cell lines with comparably high levels of specific BCL2 family proteins frequently show different responses to BH3-mimetics that target those proteins^13^. For example, there is no correlation between the abundance of MCL1 or BCL-xL and response to inhibitors that target these proteins^13^ and while BH3-profiling provides a functional measurement of the state of apoptotic signaling, actual responses to BH3-mimetics can differ from those predicted by BH3-profiling^13,14^. Collectively, these data indicate that the heterogeneous responses to BH3-mimetics in DLBCL are determined by the complex interactions between the BCL2 family of proteins and their binding partners^12,13^. Consequently, there is a need to develop better predictive tools to inform clinical decision making relating to optimal drug selection for individual patients.

Computational systems models can facilitate accurate prediction of how a molecular-scale signaling network will respond to perturbation with single cell and cell-population resolution^15,16^. Various aspects of the apoptotic signaling network have been encoded in computational models^17–32^. However, DLBCL cells exhibit variable expression of multiple anti-apoptotic BCL2 proteins and show heterogeneous expressions of both pro-apoptotic BCL2 proteins and BH3-only proteins^12,13^. Therefore, new computational models are required to capture the diverse abundances of BCL2 proteins implicated in DLBCL with their known interactional complexities to enable accurate prediction of responses to BH3-mimetics.

In this study, we aimed to establish virtual DLBCL cell lines generated from mechanistic computational models, informed by abundances of BCL2 family proteins. We aimed to use virtual cell lines to accurately predict, in silico, the experimentally-determined response of DLBCL cell lines to BH3-mimetics and identify molecular and genetic determinants of treatment resistance. Finally, we sought to establish whether a computational systems biology approach can be used to rationally predict apoptotic responses in DLBCL by computationally identifying, and experimentally validating, synergistic combinations of BH3-mimetics. We aim to lay the foundation for a personalized medicine approach to targeting the spectrum of anti-apoptotic signaling found in B cell lymphoma.

## Results

### A computational “unified-embedded-together” model enables exploration of differential sensitivities to BH3-mimetics

**Fig. 1:**
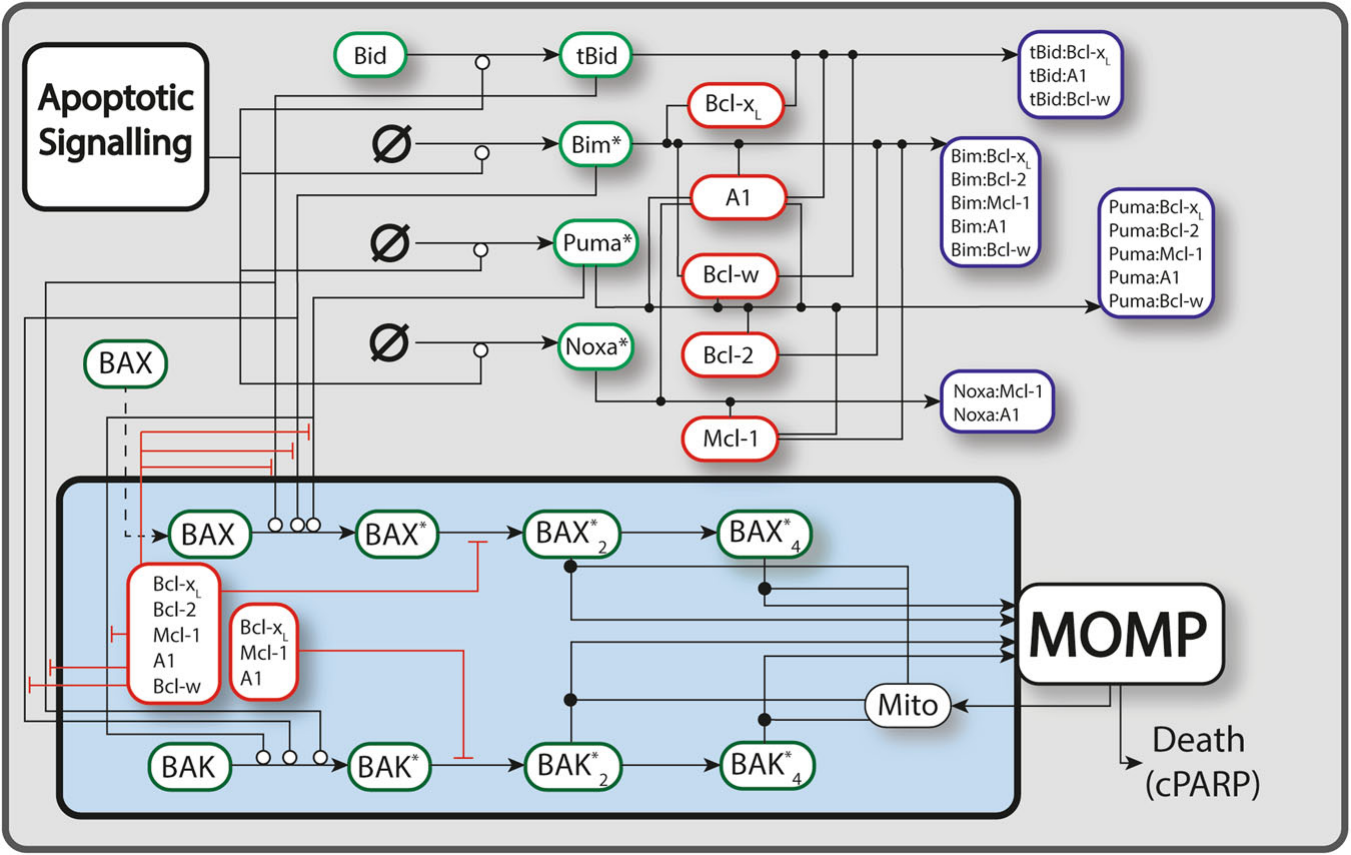
A schematic diagram of the apoptotic signaling network. Lines correspond to interactions between different species, with open circles, closed dots, and perpendicular lines denoting activation, binding, and inhibition/sequestration. Dashed lines correspond to the translocation of species. Some translocations are omitted as most BH3-only proteins and anti-apoptotic BCL2s are continuously trafficking between the cytoplasm and the MOM. Complete model details are provided in the supplemental modeling methodology and full code defining and running the model is available on GitHub (https://github.com/SiFTW/BH3Models). “Apoptotic signaling” represents any upstream activators of apoptosis, such as Fas/TRAIL activity. BCL2A1 is displayed as “A1”.

We hypothesized that the heterogeneous sensitivity of B cell lymphoma cells to BH3-mimetics is a predictable result of the state of the molecular signaling network in these cells. If true, an in silico computational model with sufficient detail and breadth would be able to predict responses, which could be validated experimentally. Combining the “embedded-together” and “unified” conceptual frameworks of apoptotic signaling (Supplementary Fig. 1A, Supplementary Note 1)^33,34^, and building upon established models of apoptotic signaling^17–32^, we constructed a computational model capturing the complex interactions between BCL2 family proteins (Supplementary Fig. 1B) with appropriate granularity to simulate the effects of BH3-mimetics (Fig. 1). We assume downstream effector-caspase induced biochemical and morphological alterations such as PARP cleavage is an inevitable consequence of MOMP. While the model does not explicitly include commonly mutated genes such as TP53 or MYC, these mutations can be simulated through their impact on kinetic rates in the signaling network.

### The experimentally-measured response to BH3-mimetics can be predicted by simulating a heterogeneous population of RC-K8 cells

**Fig. 2:**
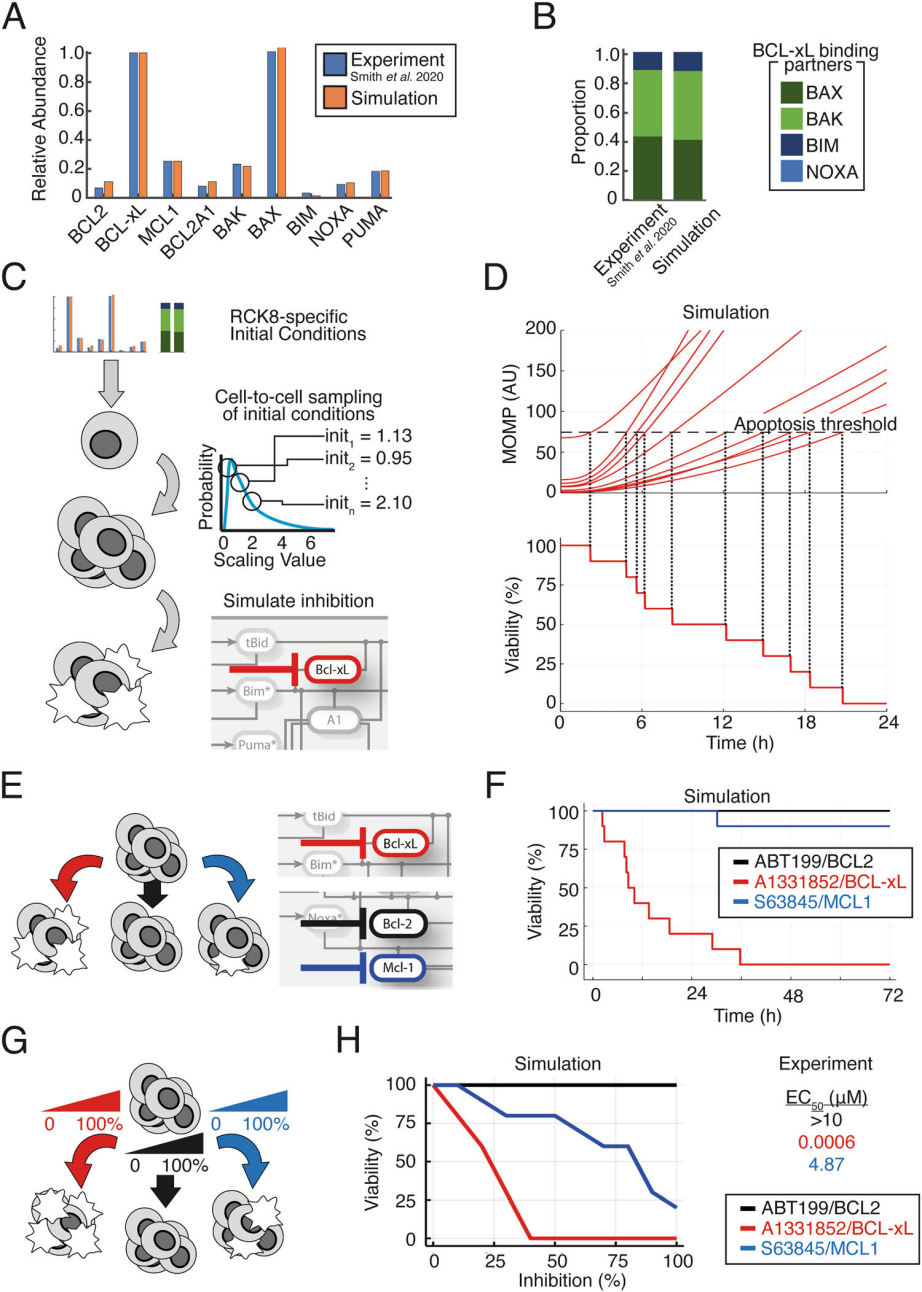
The experimentally-measured response to BH3-mimetics can be predicted by simulating a heterogeneous population of RC-K8 cells. **A** Comparison of the relative protein expression in the computational model to experimental data. Abundance of each protein is normalized to the most highly expressed anti-apoptotic BCL2 family protein and quantified from ref. ^13^. **B** Comparison of the proportion of pro-apoptotic and BH3-only BCL2 proteins bound to BCL-xL in the computational simulation compared to experimental data quantified from ref. ^13^. **C** Schematic of the method used to simulate BCL-xL inhibition in a heterogeneous cell population. From the RC-K8-speific parameterization established in panels A and B initial conditions are independently sampled from a log-normal distribution to create heterogeneous cells with distinct starting states. Within all cells in the population the target protein (e.g., BCL-xL) is inhibited and the response to this perturbation recorded (see Methods). **D** Line graphs showing the simulated response to 50% BCL-xL inhibition in a heterogeneous RC-K8 cell population. A threshold of death (10% higher than is present within the naive population) is calculated. The time of death of in each cell is determined as the time this threshold is crossed (top panel). The percentage viability of the cell population can then be determined over time in response to BCL-xL inhibition (bottom panel). **E** Schematic showing that the process used to simulate BCL-xL inhibition in **A** to **D** is repeated for multiple BH3-mimetics. **F** Line graph of the simulated viability of the RC-K8 cell population in response to BCL2 inhibition (black), BCL-xL inhibition (red) and MCL1 inhibition (blue). The viability of the cell population is recorded to 72 h to match experimental methods. **G** Schematic showing that the process used to simulate 50% inhibition in panels A to F is repeated for 10 distinct strengths of inhibition between 0 and 100% to enable comparison to dose-response experiments. **H** Line graphs showing the simulated percentage of the RC-K8 cell population viable at each percentage inhibition of the indicated target protein. This prediction can be compared to experimentally measured EC_50_ values, right^13^.

To establish the feasibility of predicting the response of DLBCL cells to BH3-mimetics we first focused on the RC-K8 cell line, chosen due to lack of response to the BCL2 inhibitor ABT-199^13^. We incorporated densitometry readings of BCL2 protein expression in RC-K8 cells measured by Western blotting into the newly established model in order to capture the expression profiles of BCL2 family proteins (Fig. 2A, see Supplementary Note 1)^13^. Interestingly, combining protein expression data with mass action kinetics using experimentally determined binding affinities revealed discrepancies in simulated heterodimer formation when compared to experimental coimmunoprecipitation data (Supplementary Fig. 2). The source of these discrepancies could include inaccuracies in protein abundance measurements, binding affinity quantification, or heterodimer abundance measurements. Alternatively, the scope of our model is restricted to one subcellular localization (the mitochondrial outer membrane), and BCL2-family proteins have been shown to localize to multiple subcellular locations outside the scope of this model^35^. We therefore incorporated densitometry readings from co-immunoprecipitation experiments, and through manual fitting of parameters representing translocation of heterodimeric complexes outside the scope of the model, we ensured the resulting computational model of the RC-K8 cell line captured BCL2-family protein abundance and heterodimerization profiles (Fig. 2A, B)^13^. Simulating the impact of BCL-xL, BCL2 and MCL1 inhibition on MOMP in this virtual RC-K8 cell line resulted in strong induction of MOMP in response to BCL-xL inhibition, weak induction of MOMP in response to MCL1 inhibition and no response to BCL2 inhibition (Supplementary Fig. 3A). This recapitulated the selective response of this line in experiments and indicated the model could predict the selective response of RC-K8 cells to BH3-mimetics.

As a fixed and reproducible fraction of the DLBCL cells undergo apoptosis in response to a given dose of BH3-mimetics^13^, we hypothesized that this may result from molecular cell-to-cell heterogeneity within the cell population. The cell-to-cell variability in the abundance of signaling components in B cells has been previously quantified through combined lineage tracing and computational modeling and was found to predictably explain fixed proportions of primary B cells undergoing distinct fates such as apoptosis, mitosis and differentiation in response to antigenic stimulation^15,16^. We therefore converted these simulations of a single average cell-population response, into simulations representing heterogeneous populations of single cells by sampling initial conditions as described previously (Fig. 2C)^15^. Simulating 50% inhibition of BCL-xL revealed that distributed initial conditions were sufficient to create heterogeneity in the timing that MOMP increased within a simulated cell population (Fig. 2D). We considered an individual cell to have died in response to BCL-xL when MOMP exceeded a threshold of 10% above the level of MOMP in the pre-treatment population and, in this way, we were able to simulate viability over time in response to BH3-mimetics in the virtual RC-K8 cells (Fig. 2D). Extending this approach to the effect of other BH3-mimetics revealed specific timings of apoptosis within the cell population, and the proportion of cells undergoing apoptosis in response to 50% inhibition of BCL2, BCL-xL and MCL1, with the largest proportion of cell death occurring rapidly in simulations of RC-K8 cells following BCL-xL inhibition (Fig. 2E and F). Simulating 0–100% inhibition of BCL2, BCL-xL, and MCL1 within a heterogeneous cell population enabled predictions of the proportion of the RC-K8 cells that would be viable at 72 h (Fig. 2G and H); these predictions were comparable to experimentally determined EC_50_ values; the concentration of a drug required to produce 50% of its maximal effect (Fig. 2H)^13^. We find that this systems biology approach predicts that RC-K8s have low EC_50_ BCL-xL inhibitors, high EC_50_ for MCL1 inhibitors, and that the EC_50_ for BCL2 inhibitors will not be reached even at 100% inhibition of BCL2 (Fig. 2H). This is in strong agreement with experimentally determined EC_50_ values (Fig. 2H right), and independent of the specific threshold of MOMP activity chosen to trigger apoptosis (Supplementary Fig. 3B). The agreement between experimentally determined EC_50_ values and simulations was an emergent property of the network state based on immunoprecipitation and co-immunoprecipitation data, as no model fitting was performed to recapitulate these EC_50_ values.

### Virtual lymphoma simulations can predict response to BH3-mimetics

**Fig. 3:**
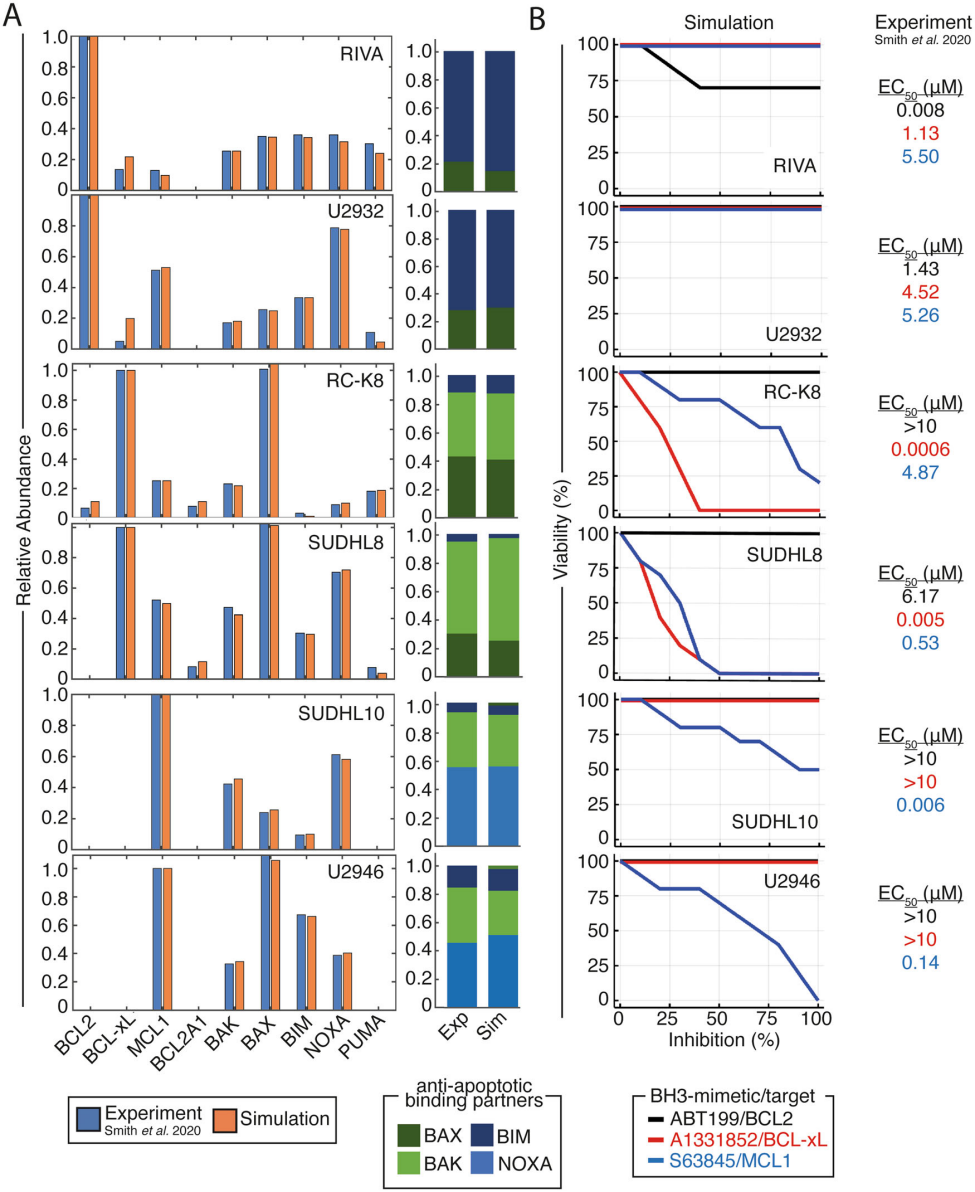
Cell line-specific computational models recapitulate experimentally measured protein expression and heterodimerisation profiles and enable accurate prediction of the optimal BH3-mimetic for each cell line. **A** Right: Comparison of the relative protein expression in the computational model to experimental data. Abundance of each protein is normalized to the most highly expressed anti-apoptotic BCL2 family protein and quantified from ref. ^13^. Left: Comparison of the proportion of pro-apoptotic and BH3-only BCL2 proteins bound to the dependent anti-apoptotic BCL2 protein in each line in the computational simulation compared to experimental data. **B** Model simulations (left) of cell population viability (%) in response to 10 different strengths of BH3-mimetics compared to experimentally-measured EC_50_ values.

As we found a systems biology approach could predict the response of RC-K8 cells to BH3-mimetics, we expanded the approach to a library of six DLBCL lines (RIVA, U2932, RC-K8, SUDHL8, SUDHL10, and U2946), chosen to capture the diversity of responses to BH3-mimetics seen within both patient samples and cell lines^13^. Of the 10 lines for which immunoprecipitation and co-immunoprecipitation data is available we randomly selected 2 lines from each annotated category of “mainly BCL2 dependent”, “mainly MCL1 dependent”, and “mainly BCL-xL dependent”. We did this to establish whether a single interaction network could capture the variety of responses seen in DLBCL cell lines. We incorporated the heterogeneous expression of BCL2 family proteins and their heterodimerization profiles, measured by immunoprecipitation and co-immunoprecipitation, in the same way as we did for RC-K8 cells (Fig. 2)^13^. The result was a library of virtual DLBCL cell lines that accurately captured the abundance and binding partners of BCL2 family proteins (Fig. 3A). Simulating the response of the library of DLBCL lines to BH3-mimetics targeting BCL2, BCL-xL and MCL1 predicted highly heterogeneous responses (Fig. 3B). Simulations predicted that RIVA cells would only respond to BCL2 inhibition, while U2932 cells were predicted to be broadly resistant to all BH3-mimetics (Fig. 3B). Comparing this prediction to experimentally determined EC_50_ values showed that the model had correctly predicted that RIVA cells only responded to BCL2-targeting ABT-199, while the predicted lack of response in U2932 cells was confirmed by the high (micromolar) EC_50_ values of U2932 cells when challenged with all three BH3-mimetics (Fig. 3B). Both RC-K8 cells and SUDHL8 cells were predicted to be most sensitive to inhibition of BCL-xL, with both lines also responding to MCL1 inhibition (at intermediate doses in SUDHL8 cells, and high doses in RC-K8 cells) (Fig. 3B). Comparing these predictions to experimentally determined EC_50_ values revealed that these computational predictions closely match experimental measurements (Fig. 3B). Simulation of SUDHL10 and U2946 cell lines predicted that these lines would only respond to inhibition of MCL1, which was validated by experimental measurements (Fig. 3B). Taken together, our virtual lymphoma simulations accurately predicted experimentally measured responses to BH3-mimetics.

### Considering genetic lesions in virtual lymphoma can improve accuracy of simulations

While the computational model could accurately assign the right drug to the right DLBCL cell lines informed by protein-expression data alone, some quantitative discrepancies between computationally predicted responses and experimentally measured EC_50_ values suggests that additional mechanisms may contribute to selective responses. Genetic lesions, such as MYC translocations and p53 mutations, are present in all modeled cell lines except RC-K8 cells^36^. Simulating the impact of commonly occurring mutations such as MYC translocation and p53 mutations on our cell lines improved the quantitative match between experiment and simulation in some cell lines (RIVA, U2932, SUDHL10, and SUDHL8) but not others (U2945, Supplementary Fig. 4). Each of these mutations can have numerous potential consequences which are likely dependent on the apoptotic network state in each cell line. We sought to predict which of these potential impacts was functionally significant in controlling the response to BH3-mimetics by computationally identifying which effect improved the match between the computational predictions and experimental validation.

RIVA cells were more sensitive to BCL2 inhibition than predicted from protein data alone (Fig. 3B). Simulating the impact of BCL2 gene amplification and MYC translocation (resulting in elevated BAX expression)^37^, increased the sensitivity of virtual RIVA cells to ABT-199 indicating an important role for these mechanisms in modulating the response to BH3-mimetics (Supplementary Fig. 5A and B). Incorporating the presence of a MYC-overexpressing subclone in U2932 cells, which resulted in increased BIM and BAX expression, explained the response of this line to high doses of ABT-199 (Supplementary Fig. 5A, B). In RC-K8 cells the magnitude of the difference in response to A1331852 and S63845 was underestimated by our simulations (Fig. 3B). The match between simulation and experiment in both lines was improved by decreasing the abundance of MCL1. Biologically, this could be mediated by the truncated p300 expressed in RC-K8 cells, which reduces the acetylation of MCL1 thereby decreasing MCL1 protein stability^38^. In SUDHL8 cells, mutated TP53 may cause the reduced gene expression of MCL1 (Supplementary Fig. 5A, B)^39^. While the simulation accurately predicted the response of SUDHL10 and U2946 cells to the MCL1 inhibitor S63845, the simulation only predicted apoptosis at high doses of the inhibitor (Fig. 3). MYC translocation in SUDHL10 may increase expression of MCL1, BIM and NOXA resulting in increased sensitivity to inhibition of MCL1, while mutated TP53 in SUDHL10 cells may decrease the affinity of MCL1 for p53 protein, increasing the binding between MCL1 and BIM (Supplementary Fig. 5A, B)^40^. Taken together these data show that by comparing computational predictions with experiment results, and iteratively improving the match between the two, we can narrow down the plethora of potential effects of genetic lesions to those that are predicted to be functionally significant. By iteratively improving the model in this way, the correlation between the predicted response from simulations and experiments across the library of virtual cell lines substantially improved (R^2^ from 0.38 to 0.67, Supplementary Fig. 5C, D). Interestingly, when assessing the impact of genetic lesions on protein expression level and heterodimerization levels, we found these remain overall unchanged and consistent with experiments, despite the large changes in dose-response to BH3-mimetics (Supplementary Figs. 6 and 7). While 4 individual protein abundances changed substantially in individual cell lines, such as MCL1, BAX and NOXA in U2932 cells, and MCL1 in SUDHL8 cells, these differences may highlight discrepancies in the experimental data or non-linearity in the relationship between immunoprecipitation bands and protein concentration (Supplementary Figs. 6 and 7). Taken together, this data suggests that while the optimum BH3-mimetic can be reliably identified from protein data alone (Fig. 3), once genetic lesions are considered, the virtual cell lines can quantitatively predict experimentally determined EC_50_ values in virtual cell lines (Supplementary Fig. 4D).

### Inherent resistance to BH3-mimetics in cellular sub-populations is the predictable result of cell-to-cell molecular variability

As fractional killing of a cell line can be explained as the predictable result of inherent molecular heterogeneity between cells (Fig. 2), we sought to use simulations to predict the molecular determinants of inherent resistance to BH3-mimetics. Simulating a dose of BH3-mimetics that causes 50% reduction in viability and then analyzing the starting state of cells revealed statistically significant differences (*p* < 0.05) in the predicted molecular network state of cells that would undergo apoptosis in response to the inhibitor when compared to those that would be resistant to the treatment (Supplementary Figs. 8–13). As expected, within a population of RIVA (Supplementary Fig. 8), U2932 (Supplementary Fig. 9), and SUDHL10 (Supplementary Fig. 10) cells responding to BCL2 inhibition, the treatment-sensitive cells had higher abundances of pro-apoptotic and lower anti-apoptotic BCL2 proteins prior to the drug being applied. Furthermore, cells that undergo apoptosis were predicted to have fewer complexes between anti-apoptotic BCL2 proteins and BH3-only proteins (Supplementary Figs 8, 9). In RC-K8 cells responding to BCL-xL inhibition (Supplementary Fig. 10) and MCL1 inhibition (Supplementary Fig. 11), the cells that undergo apoptosis expressed more pro-apoptotic BCL2 proteins, more BH3-only proteins, and more complexes between pro-apoptotic BCL2 and BH3-only proteins. Intriguingly, cells susceptible to BCL-xL and MCL1 inhibition had significantly more pro-apoptotic proteins bound to the mitochondria (Supplementary Figs. 10,11).

While BCL2A1 was not predicted to contribute to the inherent treatment-resistant cell population in some cell lines (U2932, RIVA, and SUDHL10), in others (RC-K8 and SUDHL8) the BCL-xL resistant cell populations were predicted to be significantly higher in BCL2A1 (Supplementary Figs. 10–13). These simulations predict that the complex between BAX and BCL2A1 is significantly increased, while free BCL2A1 and BCL2A1 bound to NOXA, BIM, and BID, are all significantly decreased in RC-K8 cells susceptible to BCL-xL inhibition (Supplementary Fig. 10). Interestingly, in SUDHL8 cells responding to MCL1 inhibition a significant role was predicted for BCL2A1, while this was not seen in RC-K8 cells (Supplementary Figs. 11, 13).

Virtual cell lines reveal that inherent cell-to-cell variability in the sequestration of anti-apoptotic BCL2 proteins, and subcellular localization of pro-apoptotic complexes, can create an inherent treatment resistant cell population. Importantly, we found that the mechanisms of inherent treatment resistance were predicted to be diverse between different cell lines and BH3-mimetics.

### Synergistic combinations of BH3-mimetics can be computationally identified and experimentally validated

To test the ability of this systems biology approach to enable rational targeting of DLBCL we simulated combinations of BH3-mimetics and identified a number of synergistic combinations (Supplementary Fig. 14). In BCL-xL dependent cell lines, RC-K8 (Supplementary Fig. 14C) and SUDHL8 (Supplementary Fig. 14D), synergy was predicted between BCL-xL and MCL1 inhibitors. Additionally, in RC-K8 cells, the model predicted synergy between BCL-xL and BCL2 inhibition. The exquisite sensitivity of RC-K8 cells to A1331852 monotherapy (BCL-xL-targeting BH3-mimetic) meant that experimentally testing this predicted synergy would be challenging and unlikely to identify therapeutically significant combination therapies.

**Fig. 4:**
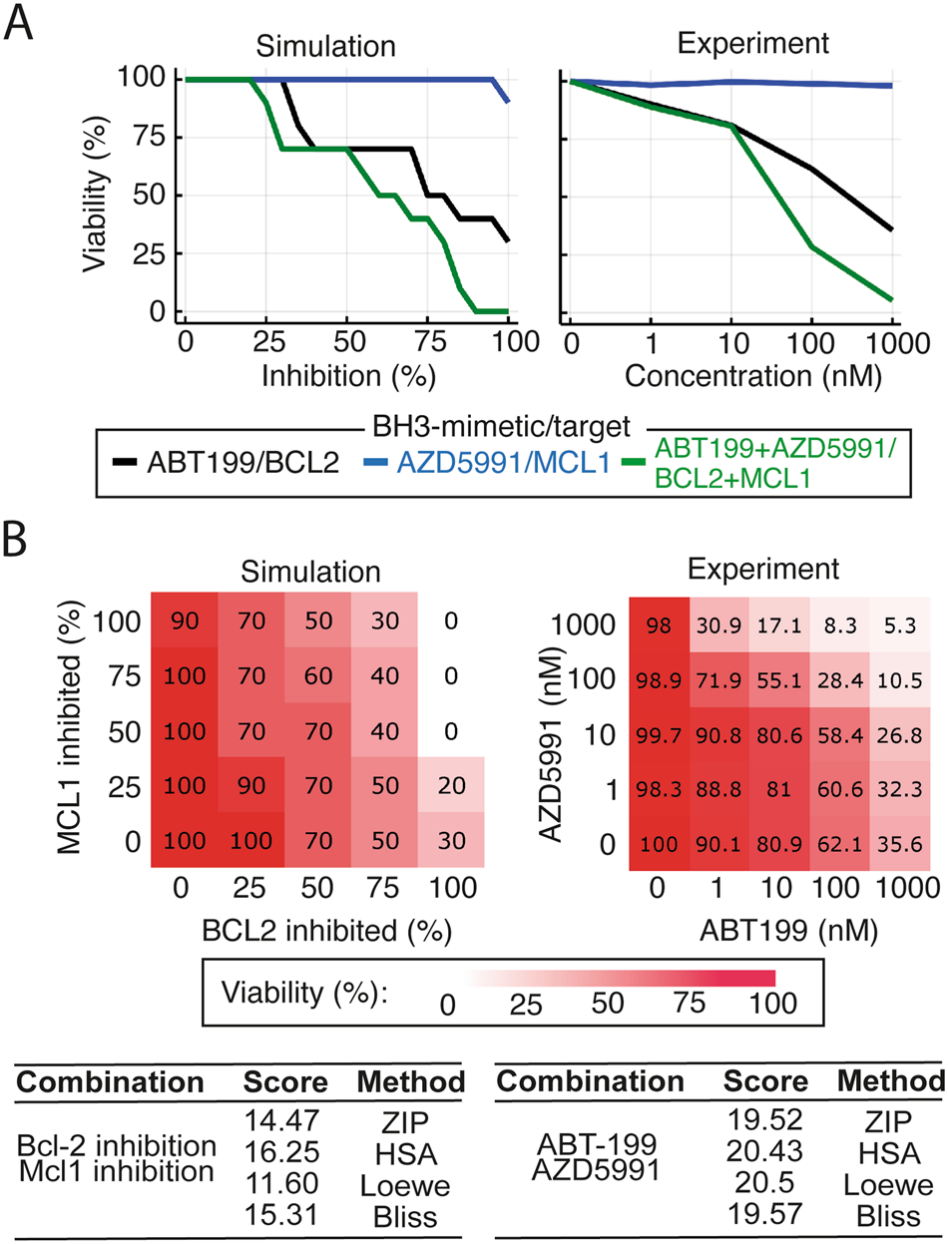
Synergistic interactions between BH3-mimetics can be computationally identified and then experimentally validated. **A** (Left panel) Model simulation predicting synergy between BCL2 and MCL1 inhibition in U2932 cells (green curve). The model predicts a moderate response to BCL2 inhibition alone (black curve), no response to MCL1 inhibition alone (blue curve) but a greater than additive response when MCL1 and BCL2 inhibition is combined at equal levels. (Right panel) Experimental data validates the model prediction, demonstrating synergy between the BCL2-specific inhibitor, ABT-199, and the MCL1-specific inhibitor, AZD5991. The combination was tested at equimolar concentrations. **B** Complete dose–response matrices showing the impact on cell viability of all combinations of simulated inhibition strengths (left) and experimentally measured BH3-mimetic doses (right) for BCL2 inhibition (ABT-199) and MCL1 inhibition (AZD5991). Viability was calculated at 72 h for both computational models and experimental measurements. Summary synergy scores of the model simulation (left panel) and experimental data (right panel), calculated using SynergyFinder software^50^.

Simulations in virtual U2932 cells predicted no response to MCL1 inhibition alone. However, adding MCL1 inhibition in the context of BCL2 inhibition was predicted to synergistically induce apoptosis (Fig. 4A, left). Predicting the impact of combining BCL2 and MCL1 inhibition across a broad range of doses indicated that it should be possible to experimentally test this prediction (Fig. 4B left, average Bliss synergy score: 15.31). The model prediction of synergy between BCL2 and MCL1 inhibitors in U2932 was experimentally tested by treating U2932 cells with ABT-199, AZD5991 and the combination in equimolar concentrations to recapitulate the computational predictions. In striking concordance with the computational prediction, U2932 cells were insensitive to the MCL1-specific monotherapy (AZD5991) but in combination with the BCL2-specific BH3-mimetic, ABT-199, showed synergistic induction of apoptosis (Fig. 4A, right). Extending this analysis to all combinations of doses in both the computational model and experimental system (Fig. 4B, Supplementary Fig. 15) confirmed the combination of BH3-mimetics was synergistic (average Bliss synergy score: 23.96). While total inhibition of MCL1 and BCL2 was predicted to reduce viability to 90% and 30%, respectively, the combination was predicted to result in a reduction to 0% viability. This was confirmed by our experimental findings, which showed that the top dose of AZD5991 and ABT-199 resulted in 98 and 36% viability respectively, while combining these two agents at the same concentrations reduced viability to 5% (Fig. 4B). Computationally identifying, and experimentally validating synergistic combinations of BH3-mimetics demonstrates that a systems biology approach has the potential to enable rational assignment of efficacious targeted therapies in DLBCL.

## Discussion

In this study we created a computational model of apoptosis and, using experimental data, we established a library of virtual DLBCL cell lines that could reliably predict experimentally measured responses to BH3-mimetics. Importantly, this systems biology approach was also capable of identifying synergistic combinations of inhibitors, which we then experimentally validated. The success of our approach when using a library of heterogeneous cell lines that captures the variability seen in patient samples indicates that this approach may form the foundation of a personalized treatment approach in DLBCL.

While the model constructed here was based on B cell lymphoma, many of these apoptotic interactions are conserved in other cell types both in health and malignancies^26,41^. To facilitate translating this approach to the challenges associated with the rational assignment of targeted inhibitors in other hematological and solid malignancies, we have made the complete library of virtual cell lines freely available (https://github.com/SiFTW/BH3Models).

One strength of computational modeling is the ability to incorporate and test the consistency of multiple modalities of data and assimilate them into a model that encapsulates the current state of knowledge for a given system. While we acknowledge that immunoprecipitation and co-immunoprecipitation blots provide semi-quantitative data, the ability of this data to create models capable of accurate predictions across a library of cell lines and BH3-mimetics indicates that our modeling approach is robust to the inherent variability of such data. While proteomic data may provide more precise quantification, this data critically lacks information on binding partners that were found to be key to determining response. The ability of computational systems biology to predict the response to BH3-mimetics motivates extending the approach to patient samples. Given the challenges of obtaining immunoprecipitation data for each patient, future work to identify surrogate biomarkers that provide clinically-obtainable measurements for the underlying network state will be required. Modeling results can be used to inform statistical regression to identify the most informative parameters that can be experimentally measured and perturbed. Regression techniques such as LASSO enable the concentrations of all molecular components from many simulations to be reduced to those most predictive of response to therapy. Previous work used this approach to computationally identify a small number of parameters that explain variability in the proliferative outcome in non-malignant B cells, which was then validated experimentally^15^. We expect that if a similar approach is taken in future work, we will be able to identify 2–3 measurable “biomarkers” that can be obtained from diagnostic samples that are predictive of which BH3-mimetic a particular tumor is most vulnerable to. This would enable models to be created that represent patient’s tumors using measurements that can feasibly be obtained from diagnostic samples, potentially unlocking more personalized treatments for DLBCL.

The model utilized here is a sub-network of the apoptotic signaling network. As such, the model only accounts for the mutational and molecular heterogeneity in a proportion of the important signaling networks implicated in B cell malignancies. To assess the impact of dysregulation of multiple signaling networks (including upstream receptor proximal signaling, NF-κB regulation, cell cycle and differentiation), and treatment modalities targeting these networks, future work will necessarily require more comprehensive models. Established multi-scale B cell models containing these signaling networks and linking them to cell fates have demonstrated utility in predicting the emergent impact of genetic events and inhibitors on cell population phenotypes^15,16^. Re-purposing these models for lymphoma, and incorporating the insight generated here, may enable rational targeting of additional therapies and combinations of therapies that target these signaling networks (such as ibrutinib^42^, idelalisib^43^, and copanlisib^44,45^).

While we have tested many of the computationally generated predictions in this study in order to validate our models, many intriguing predictions remain as avenues for future work. For example, predictions of which cells will survive BH3-mimetic treatment may provide insight into how combination therapies or pre-treatments might be deployed to optimize the molecular network state for sensitivity to BH3-mimetics. It is likely that treatment-resistant cells are vulnerable to alternative targets as each apoptotic network state we investigated had both resistances and vulnerabilities. The cell lines we investigated covered a broad range of expression of BCL2 family member expression, a broad variety of protein-protein interaction states, multiple cell-of-origin classifications, and common mutational events (Fig. 3 and Supplementary Fig. 4). However, the existence of B cell lymphoma lines (such as DLBCL line HBL-1, and primary mediastinal B cell lymphoma line such as Karpas-1106) that do not respond well to any BH3-mimetic indicates that additional cellular archetypes exist^13^. Identifying therapeutic vulnerabilities in the network state of these broadly resistant cells may help target the most challenging cases. However, as these lines are in the minority, the primary challenge in adopting more targeted therapies in B cell lymphoma appears to be getting the right combination of drugs that are already available into the right patients. Systems biology modeling may enable this and by modeling individual patients unlock the potential of personalized medicine.

## Methods

### Experimental procedures

U2932 cells were maintained in RPMI-1640 media (21870076, Gibco; Life Technologies) with 10% fetal calf serum (10270-106, Gibco). Cells were plated at 4 × 10^5^ cells/mL and treated with ABT-199 and AZD5991 (Selleck Chemicals, Houston, TX^46^,) 24 h before analysis using CellTiter-Glo viability assay (Promega, Madison, WI). Response was normalized to a DMSO control.

### BCL2 family signaling network model topology

The BCL2 family signaling network model was constructed from known protein interactions, requiring expression and degradation parameters for each protein^29,32,47^, as well as binding rates for interacting proteins (Supplementary Table 1)^29,32^. The chosen set of interactions yields our BCL2 network topology (Fig. 1). Additional description of the conceptual frameworks and approach used in model construction are provided in Supplementary Note 1.

### Model construction

Model construction was achieved by building on previous models and incorporating experimentally measured BCL2 family protein expressions, binding affinities, kinetic rates, and knowledge of genetic lesions, see Supplementary Note 1^13,32,47^. The model contains 80 molecular species, represented by 80 ODEs, 202 reactions, and 203 parameters, which are described in Supplementary Note 1. All parameters are consistent for all simulations (Supplementary Table 1), other than expression/degradation rates indicated in Supplementary Table 2. These cell line-specific parameters were manually fit to recapitulate cell line-specific IP and co-IP data^13^. Where a particular cell line does not contain detectable protein by IP or co-IP we exclude that protein, and complexes containing that protein, from the ODE systems to reduce computational complexity. Parameter fitting was performed manually, informed by experimental data as described in Supplementary Note 1.

Three*.csv* files were created for each virtual cell model, containing reactions between interacting proteins, the rate laws defining the reactions and a parameter file, respectively. Reactions between interacting proteins are governed by mass action kinetics, where interactions can be either simple binding and unbinding or binding/unbinding leading to activation of a new species. The three*.csv* files were used as inputs, to generate the system of Ordinary Differential Equations (ODEs) that defines our network model using custom python code (available: https://github.com/SiFTW/CSV2JuliaDiffEq).

### Solving models/running simulations

The programmatically generated model files were imported into Julia^48^, and solved using the DifferentialEquations.jl package^49^. Numerical simulations, initial conditions, solver options, and the code to generate all figures are available on GitHub (https://github.com/SiFTW/BH3Models).

### Simulating cell-to-cell variability

Cell populations of 100 cells were simulated with molecular cell-to-cell variability introduced in initial conditions. Initial conditions were distributed using a lognormal distribution with coefficient of variation (32%) defined by previous live-cell lineage tracking experiments in primary B cells^15^.

### Reporting summary

Further information on research design is available in the Nature Portfolio Reporting Summary linked to this article.

## Supplementary information

Supplementary Material

Reporting Summary checklist

## Data Availability

All data used to inform the model are available in the GitHub repository, experimental data are available from the authors. https://github.com/SiFTW/BH3Models.

## Code availability

All code used in the study are available in the GitHub repository: https://github.com/SiFTW/BH3Models. https://github.com/SiFTW/CSV2JuliaDiffEq.
