# Supplementary material for: Computational modeling of DLBCL predicts response to BH3-mimetics
Source: NPJ Syst Biol Appl. 2023 Jun 6;9:23. doi: 10.1038/s41540-023-00286-5 (PMC10244332; doi:10.1038/s41540-023-00286-5)
Supplement: Supplementary file 1 — Supplementary Material [file 41540_2023_286_MOESM1_ESM.pdf]

Supplementary Note 1

Parameter Fitting

Expression and degradation parameters and binding and unbinding rates for the network model were taken from published results including computational models developed for other biological contexts.[1-3] Cell line-specific BCL2 protein expression parameters were manually adjusted to recapitulate experimentally measured protein expression profiles in each cell line.[4] Experimentally measured expression and dimerization profiles were quantified using ImageJ.[5] The emergent heterodimerization profiles were compared to experimentally measured dimerization profiles.[4] To address discrepancies between simulated heterodimer profiles and experimentally measured heterodimerization profiles, we assume that BCL2 protein homodimers and heterodimers are localized in multiple locations across the cell, including the endoplasmic reticulum, the Golgi apparatus and the nucleus, and that biochemical measurements of protein expression will account for all the proteins regardless of location. However, we only model BCL2 proteins in the cytoplasm and MOM-bound. Therefore, we accounted for complexes trafficked to subcellular localizations outside of the model's scope by manually altering the degradation rates of protein complexes within the model's signaling network. Upon establishing accurate protein expression and binding profiles for each line's parameters, these were kept consistent for all subsequent simulations, other than those influenced by genetic lesions (see Supplementary Table 1).

The conceptual frameworks used in modelling

Multiple conceptual models/frameworks of the complex interaction between BCL2 family members have been proposed, including the direct activation model, the displacement or de-repression model, embedded-together model and the unified model.[6-16] A review of these conceptual frameworks is given in Shamas-Din *et al.*[17] We consider both the embedded-together and unified models, which introduce a role for the mitochondrial membrane, as MOMP does not ensue until BAX and BAK achieve their active conformation in the membrane. Furthermore, in both these models, anti-apoptotic BCL2 proteins sequester BH3-only proteins and pro-apoptotic BCL2 proteins. However, a distinguishing feature of the unified model is that inhibition of apoptosis via sequestration of pro-apoptotic BAX and BAK is more efficacious than inhibition via

sequestration of BH3-only proteins. To capture the range of potential interactions in B cell lymphoma cells, we encoded features from both conceptual models, along with previously published models, and recently published experimental data into our computational model (Figure 1A).[1, 3, 7, 11, 18-31] Briefly, we consider inactivator or sensitizer BH3-only proteins to interact exclusively with anti-apoptotic BCL2 proteins impeding the translocation of anti-apoptotic BCL2 to the mitochondrial membrane and the subsequent interaction of anti-apoptotic BCL2's with pro-apoptotic BAX and BAK and activator BH3-only proteins.[9, 12, 32] While we consider activator BH3-only proteins to perform the dual function of directly activating pro-apoptotic BCL2 proteins, initiating membrane permeabilization and also sequestering anti-apoptotic BCL2 proteins, inhibiting their interaction with pro-apoptotic BCL2 proteins.[15, 16]

Having established the conceptual framework, we then encoded knowledge of the selective interactions between BCL2 family proteins. We considered that all anti-apoptotic BCL2 proteins interact with pro-apoptotic BAX through their conserved BH3 binding groove, while, among the anti-apoptotic BCL2 family, only BCL-xL, MCL1 and BCL2A1 interact with pro-apoptotic BAK, also through their conserved BH3 binding groove.[10, 13, 16, 33-36] We considered that BH3-only proteins bind to anti-apoptotic BCL2 proteins in the same binding groove but with greater selectivity for binding partners. BIM and PUMA interact with all anti-apoptotic BCL2 proteins, while BID or its truncated variant tBID binds to BCL-xL, BCL2A1 and BCL-w, and Noxa binds selectively to MCL1 and BCL2A1. Furthermore, BID (tBID), BIM and PUMA but not NOXA are known to activate both BAX and BAK, resulting in BAX and BAK binding to the mitochondrial membrane and MOMP (Figure 1 and Supplementary Figure 1).[6, 16, 17, 33-37]

The BCL2 family of proteins is classified into three groups: pro-apoptotic BCL2 proteins, which interact directly with the mitochondrial outer membrane, permeabilizing the membrane; BH3-only proteins (BH3s), which directly or indirectly activate pro-apoptotic BCL2 proteins; and anti-apoptotic BCL2 proteins, which directly or indirectly inhibit pro-apoptotic activation and pore formation.[17] Adding an extra layer of complexity, interactions of BCL2 proteins are regulated by which family members are present, their relative binding affinities and concentrations, as well as their spatial localization.

In this work, inactivator or sensitizer BH3s interact exclusively with anti-apoptotic BCL2 proteins impeding the translocation of anti-apoptotic BCL2 to the mitochondrial membrane and the subsequent interaction of anti-apoptotic BCL2s with pro-apoptotic BAX and BAK and activator BH3s.[9, 12, 32] Activator BH3s perform the dual function of directly activating pro-apoptotic BCL2 proteins, initiating membrane permeabilization and also sequestering anti-apoptotic BCL2 proteins, inhibiting their interaction with pro-apoptotic BCL2 proteins.[15, 16]

In Figure 1, Apoptotic signaling acts as a proxy for the extrinsic apoptotic stimuli and the caspase network and, thus, activates the BH3s in the model. The model consists of two compartments, the cytoplasm (grey shaded region) and the mitochondrial outer membrane (MOM) (blue shaded region). Lines in the diagram denote interactions between species, with open circles corresponding to an activating interaction, closed dots corresponding to a binding interaction and perpendicular lines corresponding to inhibition/sequestration.

Experimental studies have demonstrated how, when exposed to extrinsic stimuli, cells undergo a protracted and variable delay preceding caspase activation (preceding BH3-only activation in this model), but a swift evolution to MOMP once activation is initiated.[38, 39] Similar “variable-delay, snap-action” switching behavior seen experimentally has been observed in previous models of apoptosis.[1, 3, 27] Our variable of interest in the model is MOMP, which is a proxy for the proportion of PARP that has been cleaved and, therefore, also a proxy for cell death. Supplementary Figure 3 (A) shows the result of our model simulation for the level of MOMP as a function of time for different apoptotic signaling doses (labelled Signal in the figure). As seen in both experimental data and computational models, the rate of increase of MOMP increases with increasing doses of the apoptotic signal. However, MOMP saturates at a higher level in our model as the apoptotic signal increases. In previous models, the proportion of cleaved PARP (cPARP) is used as the response variable and appears to reach the same saturation level for different doses of extrinsic death. One possible explanation is that the positive feedback from MOMP via caspase-3 and caspase-8 results in a threshold level of MOMP generating more MOMP. Since we have omitted the caspase network in our model, it is reasonable to assume that our system is equivalent to previous systems, including the positive feedback mechanism. Thus, MOMP is a legitimate proxy for cPARP and cell death.

BH3-mimetics function to sequester anti-apoptotic BCL2 proteins, releasing pro-apoptotic BAX and BAK. However, in our model simulations, we model BH3-mimetic activity as anti-apoptotic BCL2 protein inhibition. Supplementary Figure 3 (A) shows the response to anti-apoptotic BCL2 family inhibitors our model (20 and 50% inhibition).

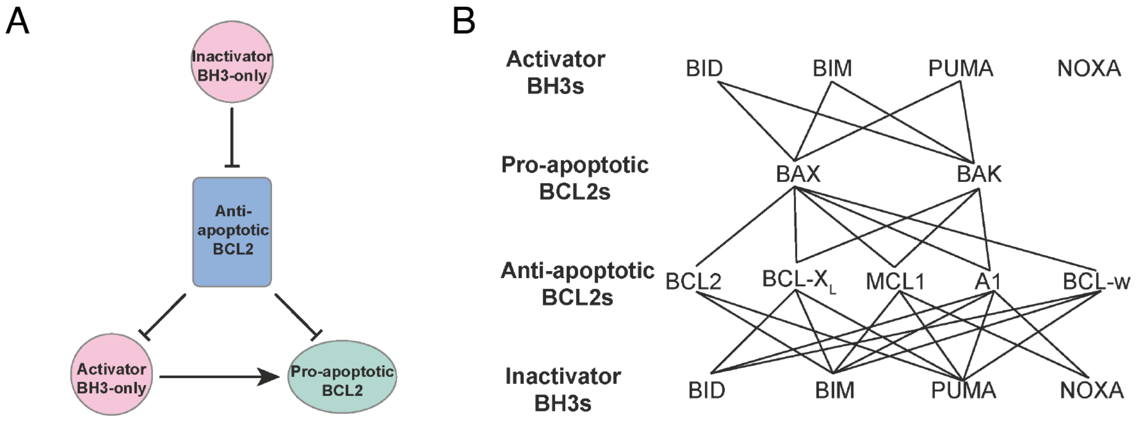

Supplementary Figure 1. A unified-embedded-together model of apoptosis. (A) Schematic diagram of the unified-embedded-together model. (B) Graph of the BCL2 family of protein interactions. Nodes correspond to BCL2 family members, while links indicate interactions between corresponding proteins.

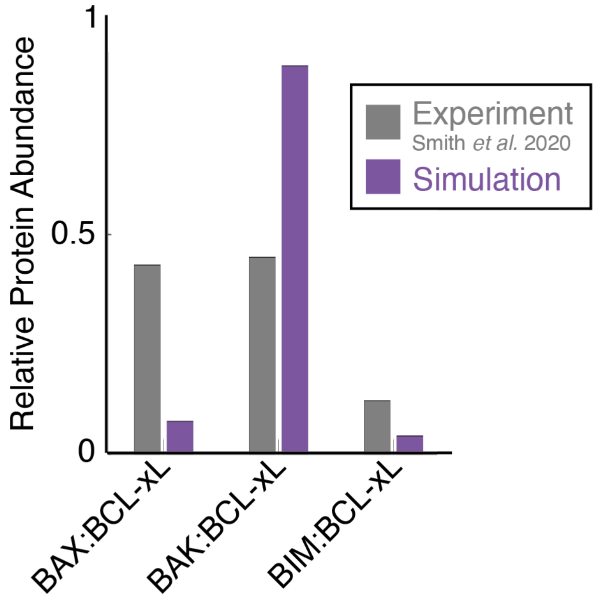

Supplementary Figure 2. Heterodimerization profile in RC-K8 cells that emerges from protein expression profile in Figure 1A in both experimental data (grey[4]) and model simulations (purple). Expression values are normalized to show proportion of BCL-xL bound to BAX, BAK and BIM.[4]

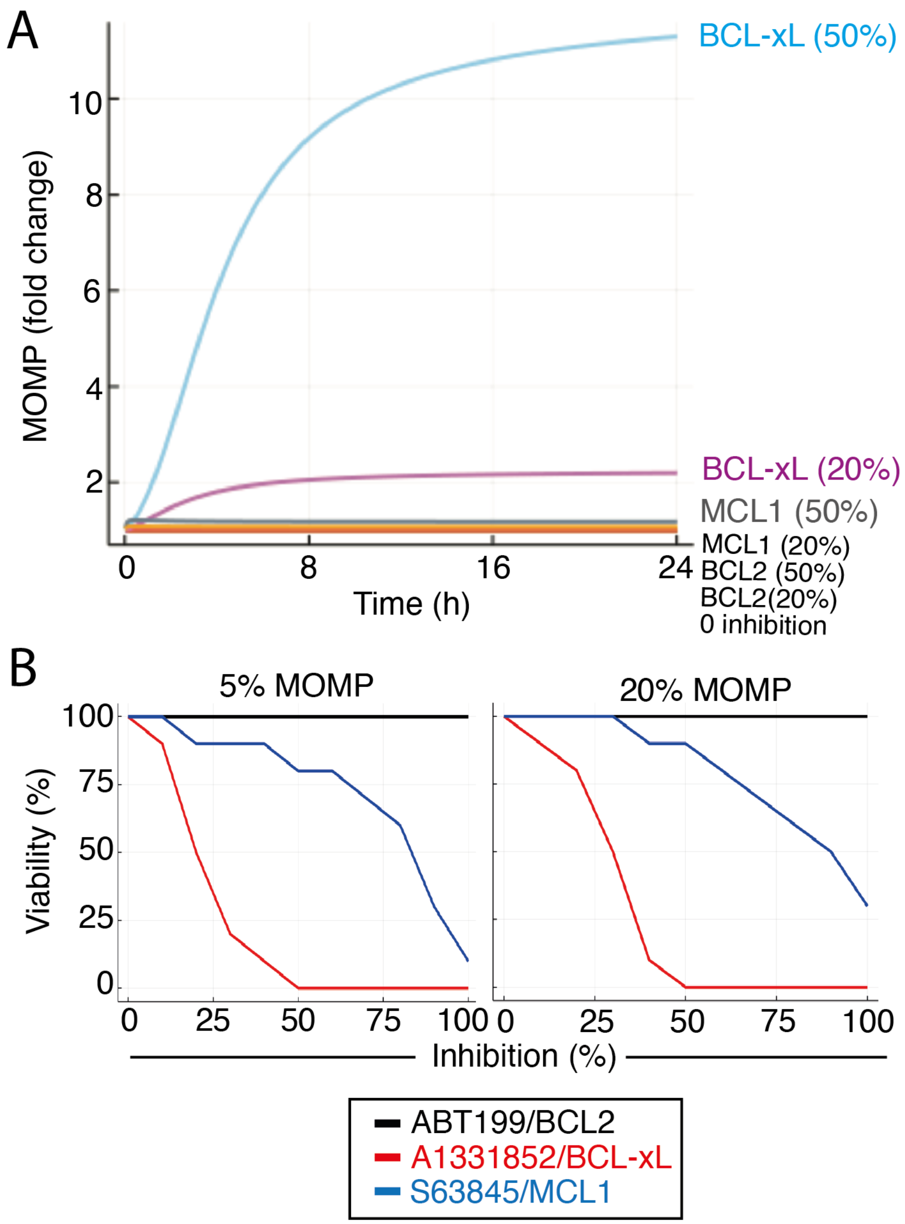

Supplementary Figure 3. (A) RC-K8 specific response to BH3-mimetics. The model simulation displays the level of MOMP achieved in response to the indicated level of inhibition of the indicated BCL2 family protein. The level of MOMP is measured as a fold change from the basal level of MOMP (MOMP level before administering the BCL2 inhibitor). (B) The viability (%) in response to inhibition of the indicated BCL2 family proteins for the respective virtual cell lines with MOMP threshold decreased 2x from the value used in Figure 2 (left, 5% MOMP inhibition), and increase 2x from the value used in Figure 2 (right, 5% MOMP inhibition).

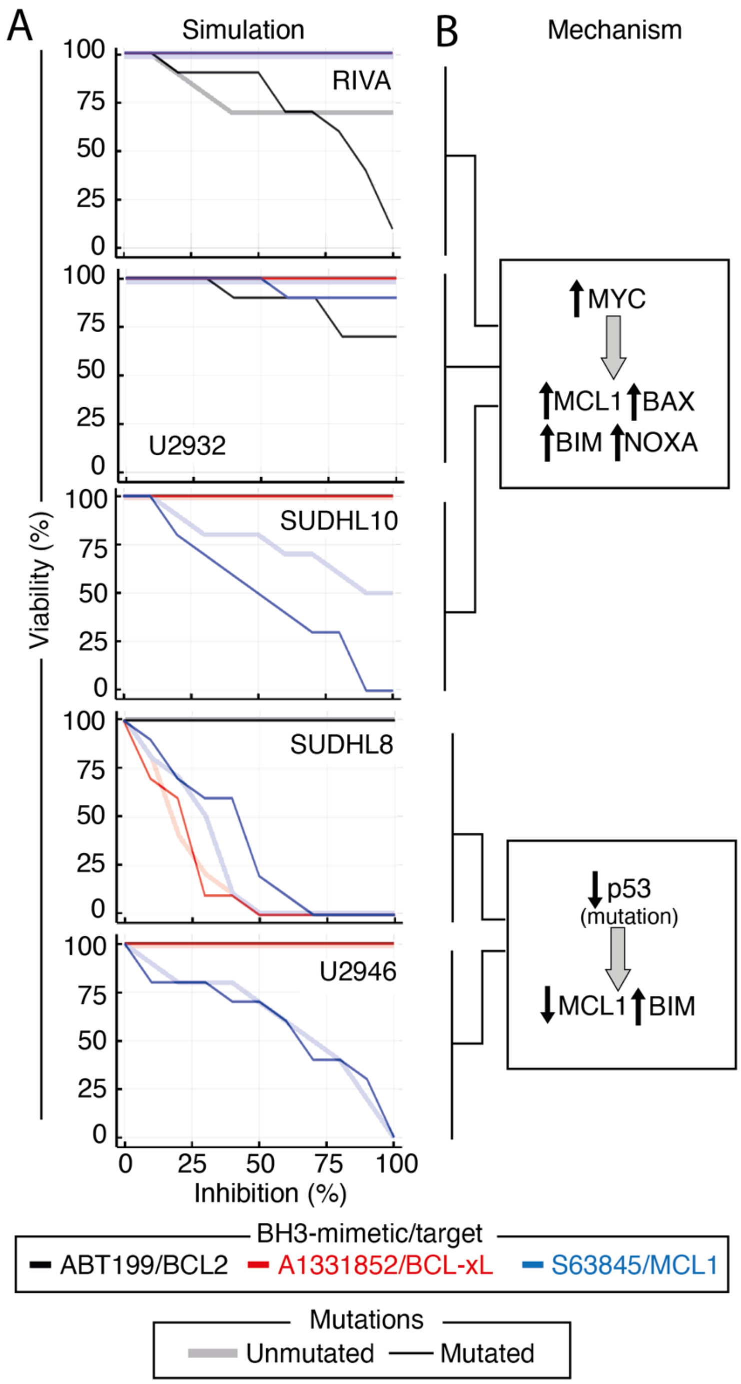

Supplementary Figure 4. (A) The viability (%) in response to inhibition of the indicated BCL2 family proteins for the respective virtual cell lines (Figure 3) modified to consider the mutational profile of the cells. The original survival curves from Figure3 informed by protein expression alone are indicated with faint lines. Black, red and blue curves are the response corresponding to BCL2, BCL-xL and MCL1 inhibition, respectively. (B) The proposed mechanism that was simulated to improve the match between experimentally measured EC_{50} values and the model prediction.

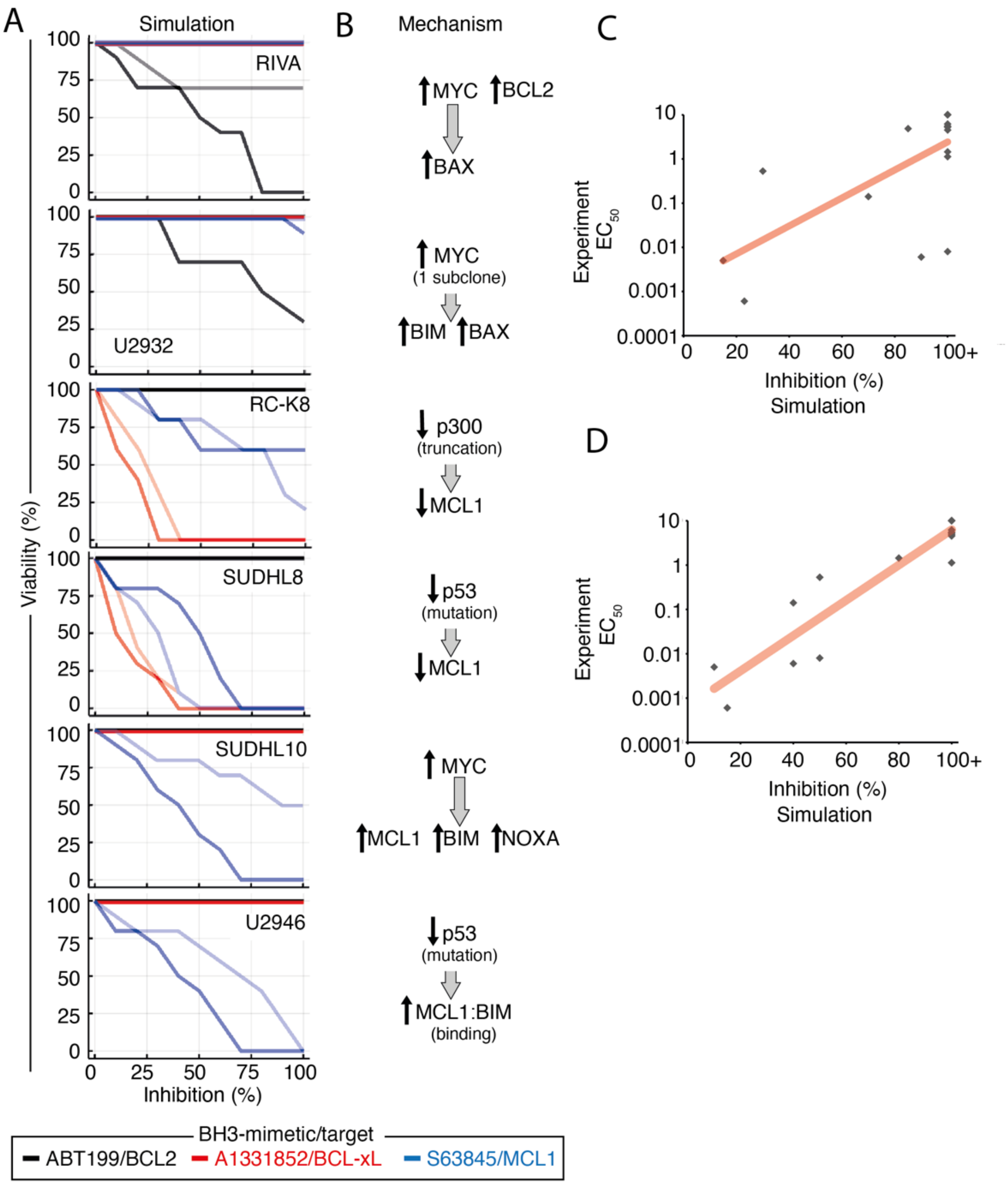

Supplementary Figure 5. (A) The viability (%) in response to inhibition of the indicated BCL2 family proteins for the respective virtual cell lines (Figure 3) modified to consider the mutational profile of the cells. The original survival curves from Figure 3 informed by protein expression alone are indicated with faint lines. Black, red and blue curves are the response corresponding to BCL2, BCL-xL and MCL1 inhibition, respectively. (B) The proposed mechanism that was simulated to improve the match between experimentally measured EC_{50} values and the model prediction. (C and D) Computationally simulated inhibition percentage where 50% viability is predicted, compared to experimentally measured EC_{50} values in virtual cell lines (C, R^2=0.38) and virtual cell lines modified to consider the mutational profile of the cells (D, R^2=0.67).

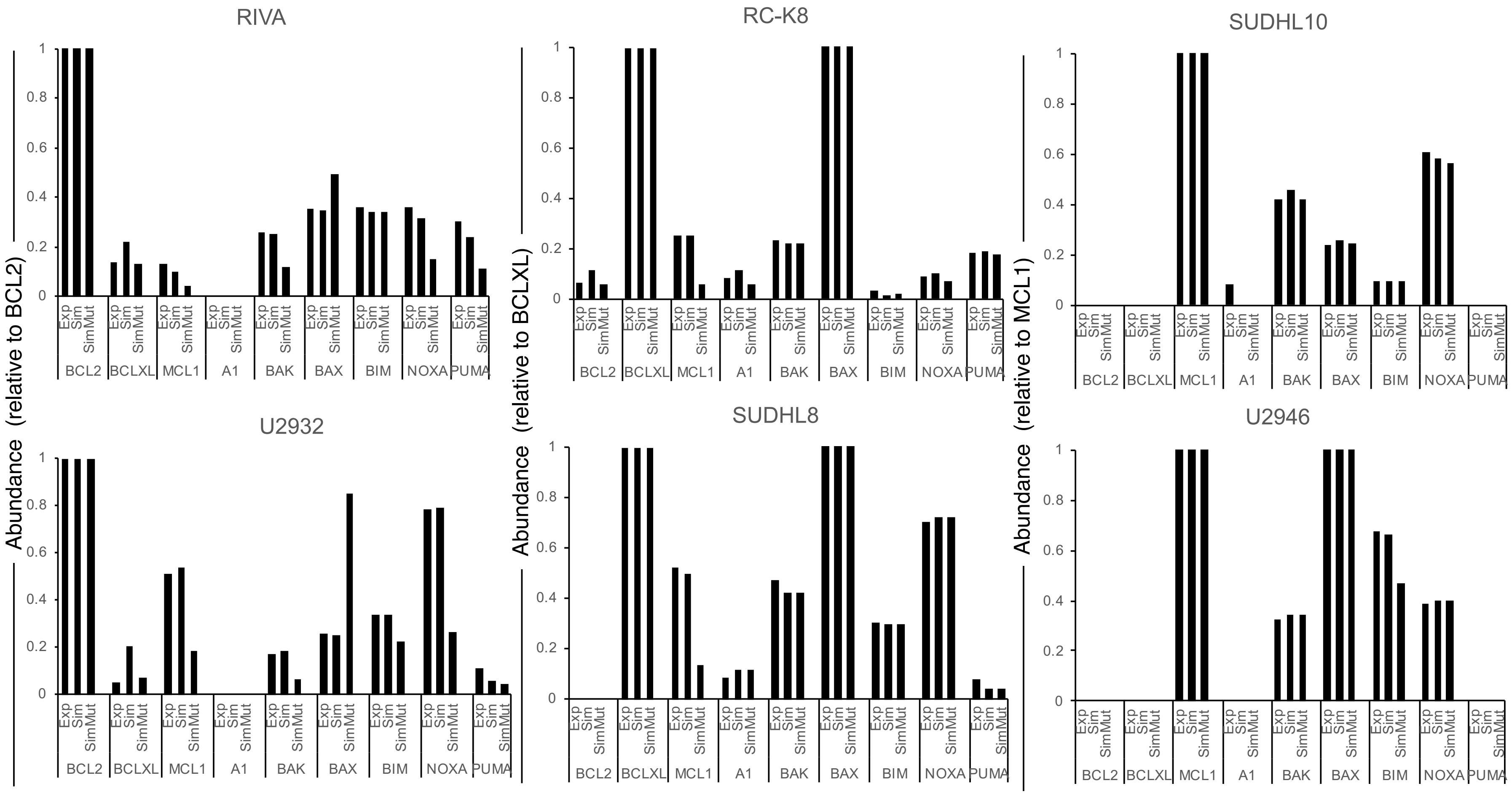

Supplementary Figure 6. Abundance of the indicated species normalized to BCL2 (left) BCL-xL (middle) and MCL1 (right) in the indicated cell lines. Exp = experimental data, Sim = simulation based on experimental abundance data alone. SimMut = simulation after adjusting parameters to account for the mutations described in Supplementary Figure 5.

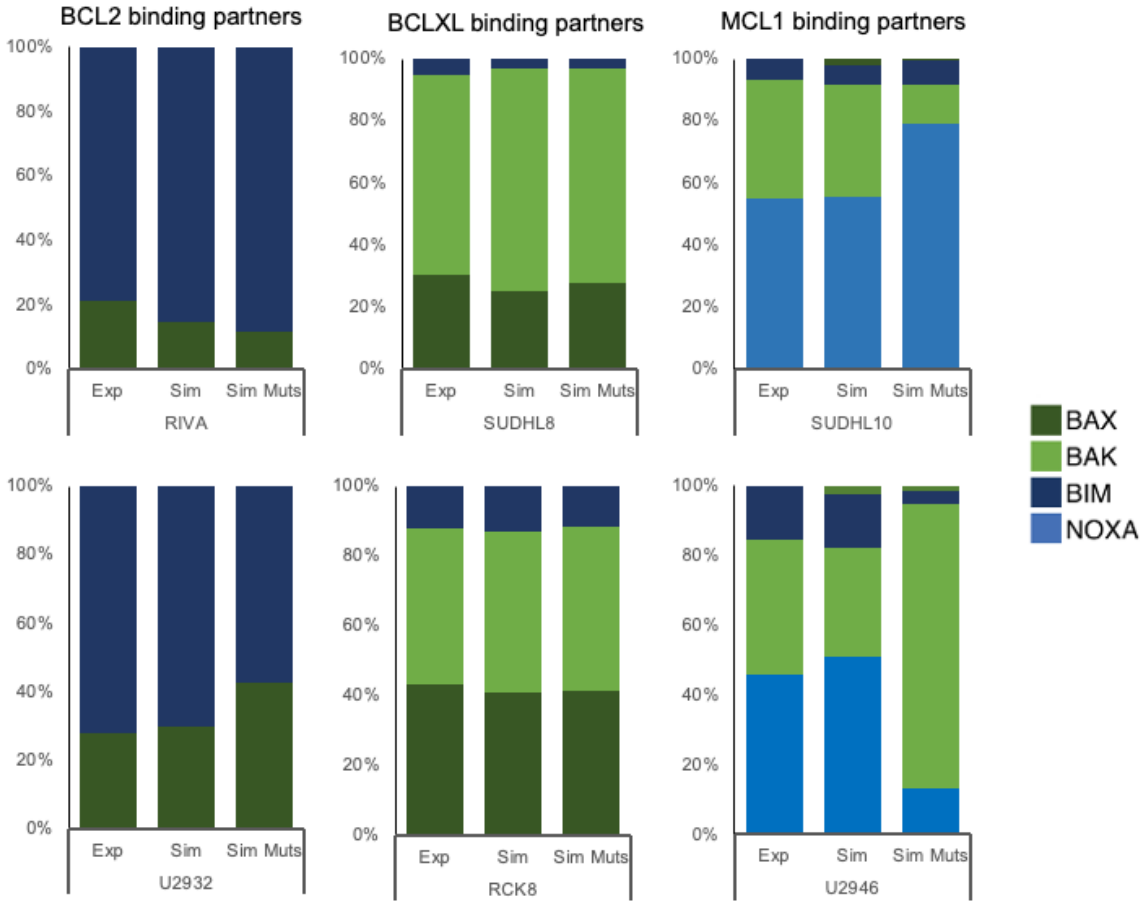

Supplementary Figure 7**.** Binding partners of BCL2 (left), BCLXL (middle) and MCL1 (right), in the indicated cell lines. Exp = experimental data, Sim = simulation based on experimental abundance data alone. SimMut = simulation after adjusting parameters to account for the mutations described in Supplementary Figure 5.

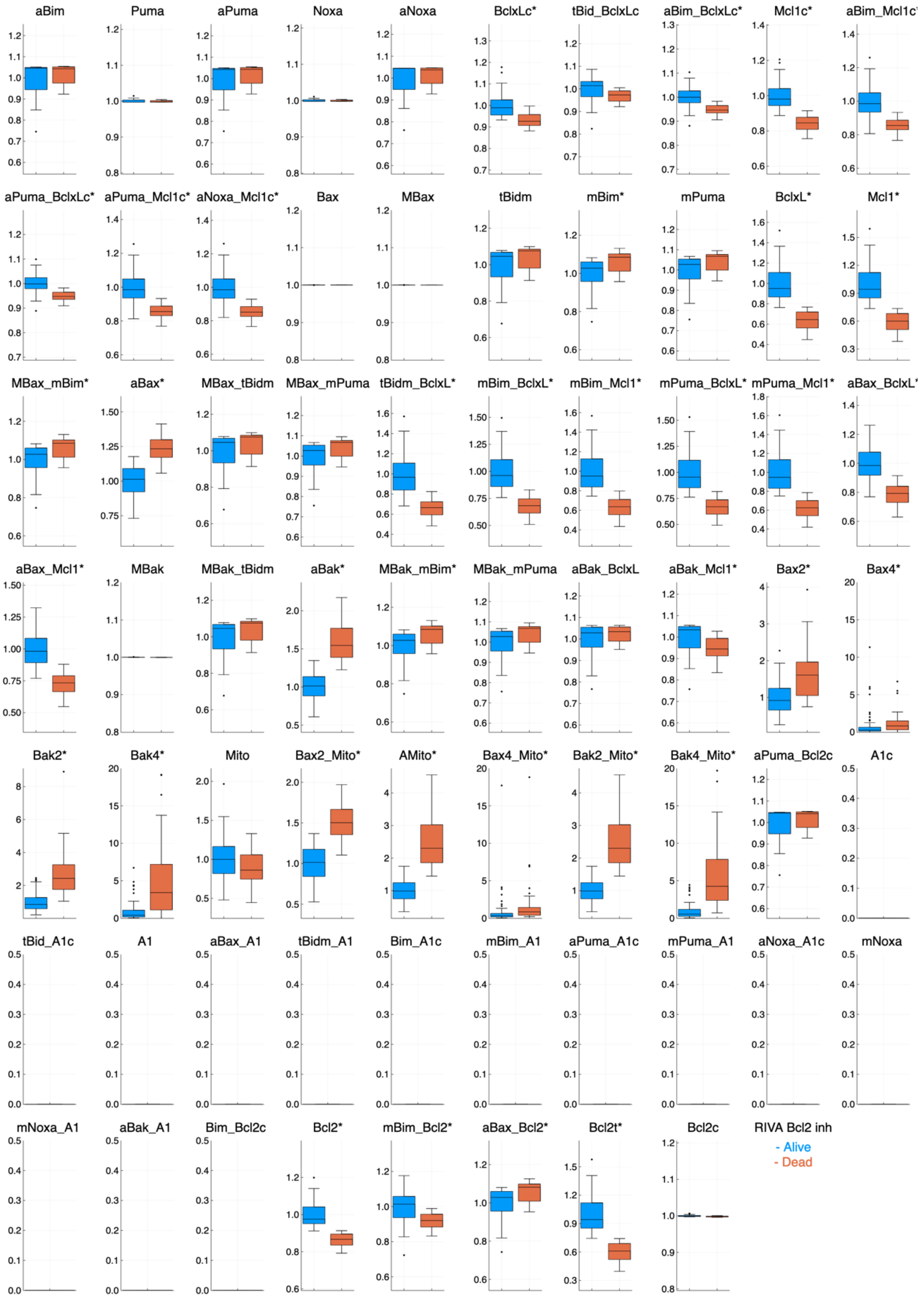

Supplementary Figure 8. Differences in initial protein abundances in RIVA cells between cells that survive BCL2 inhibition (Blue box) and cells that undergo death (Red box). * = p<0.05 by two-sample KS test. Median is indicated by a line, with the box extending to the first and third quartile. Whiskers indicate the range of the data with suspected outliers plotted individually.

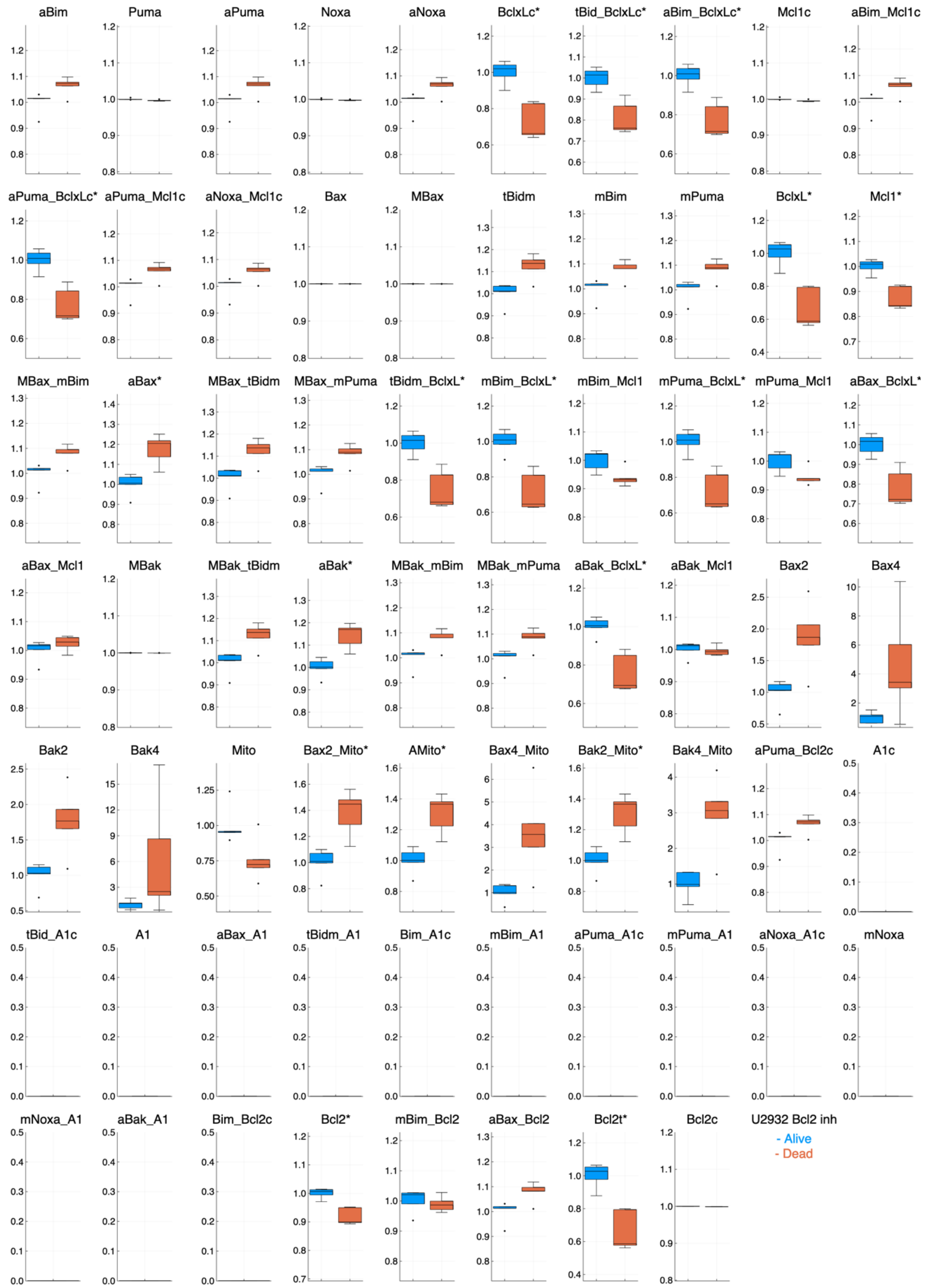

Supplementary Figure 9. Differences in initial protein abundances in U2932 cells between cells that survive BCL2 inhibition (Blue box) and cells that undergo death (Red box). * = p<0.05 by two-

sample KS test. Median is indicated by a line, with the box extending to the first and third quartile. Whiskers indicate the range of the data with suspected outliers plotted individually.

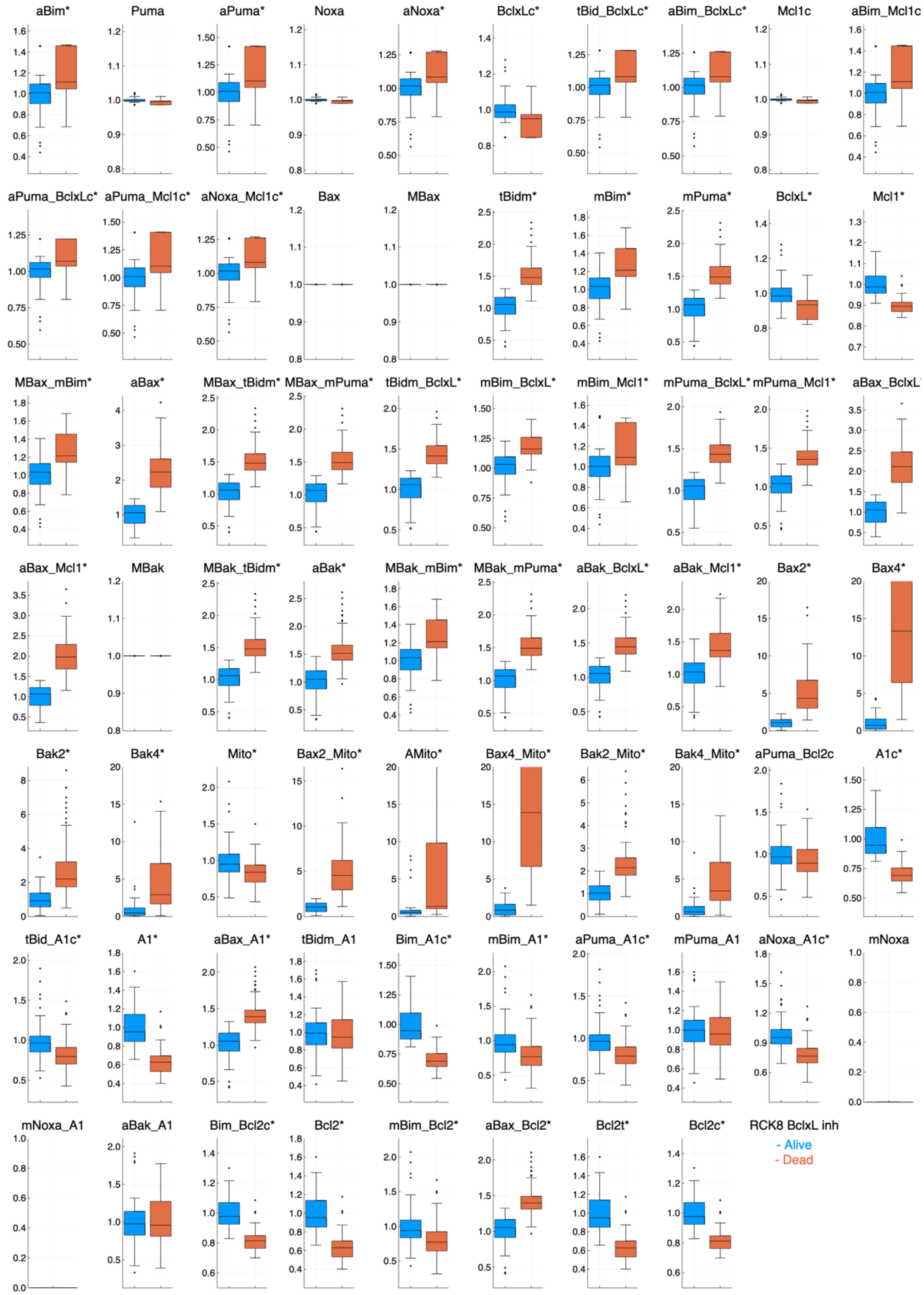

Supplementary Figure 10. Differences in initial protein abundances in SUDHL10 cells between

cells that survive BCL-xL inhibition (Blue box) and cells that undergo death (Red box). * = p<0.05 by two-sample KS test. Median is indicated by a line, with the box extending to the first and third quartile. Whiskers indicate the range of the data with suspected outliers plotted individually.

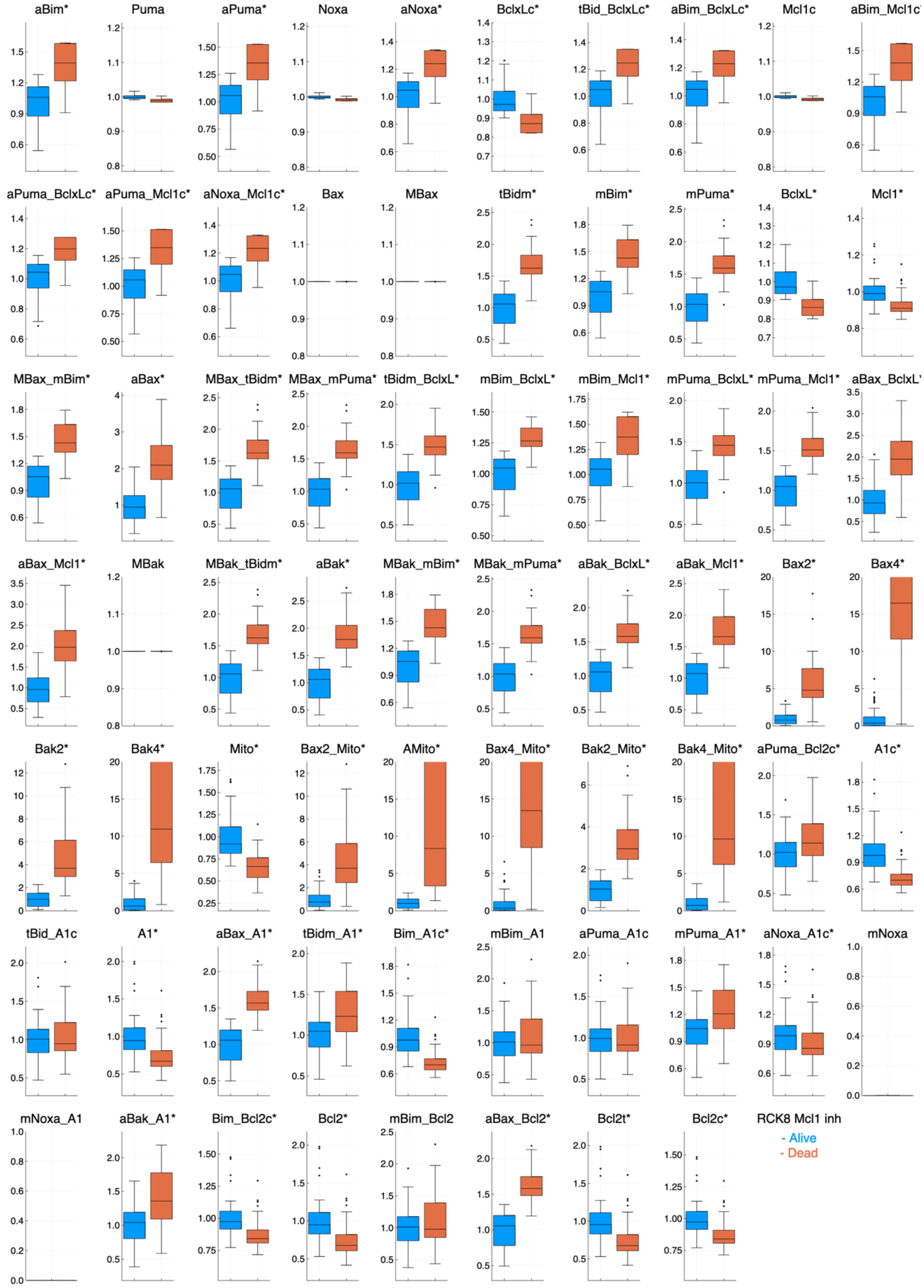

Supplementary Figure 11. Differences in initial protein abundances in RC-K8 cells between cells

that survive (Blue box) and cells that undergo death (Red box) in response to MCL1 inhibition. * = $p<0.05$ by two-sample KS test. Median is indicated by a line, with the box extending to the first and third quartile. Whiskers indicate the range of the data with suspected outliers plotted individually.

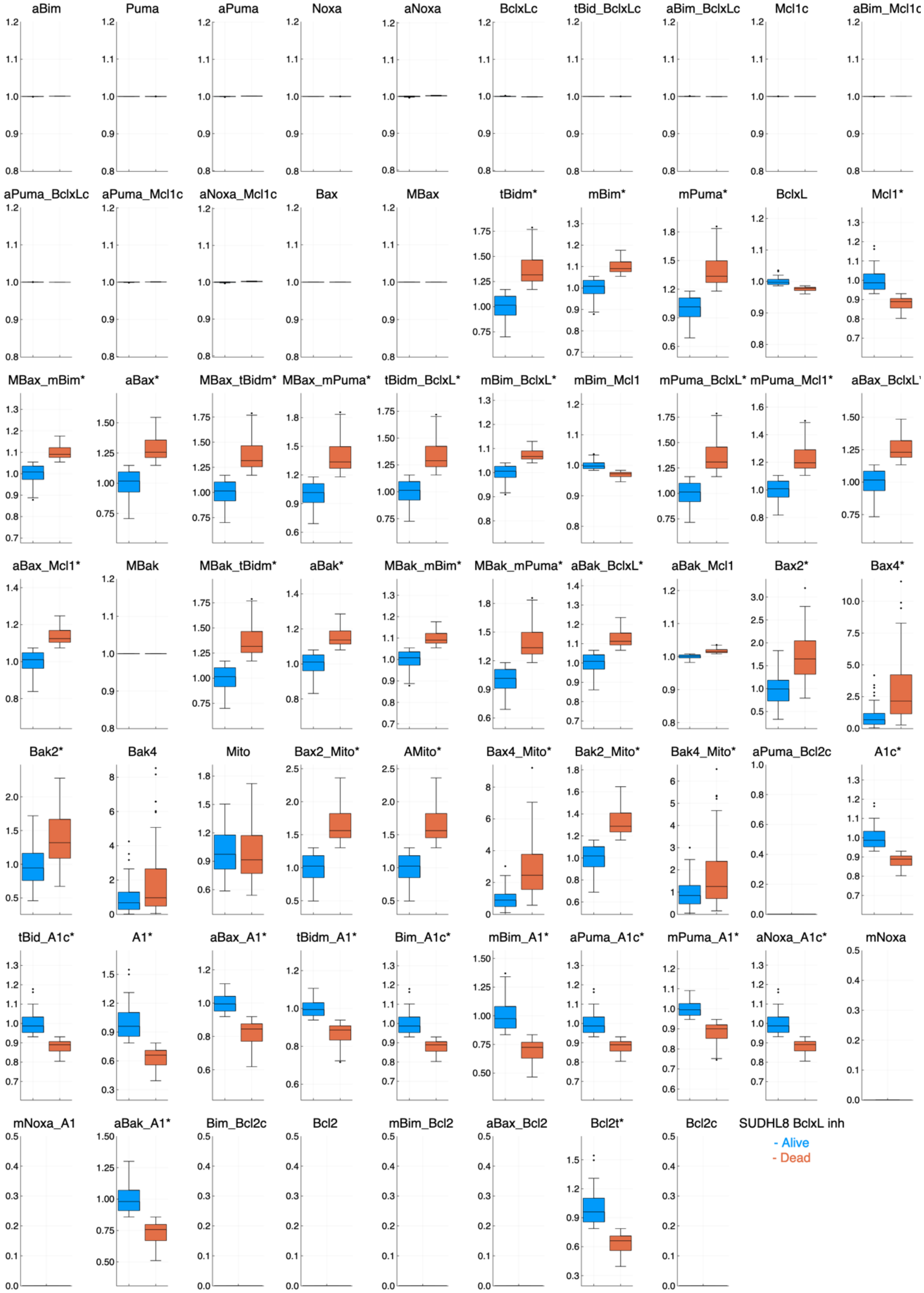

Supplementary Figure 12 Differences in initial protein abundances in U2946 cells between cells that survive (Blue box) and cells that undergo death (Red box) in response to BCL-xL inhibition. * = p<0.05 by two-sample KS test. Median is indicated by a line, with the box extending to the first

and third quartile. Whiskers indicate the range of the data with suspected outliers plotted individually.

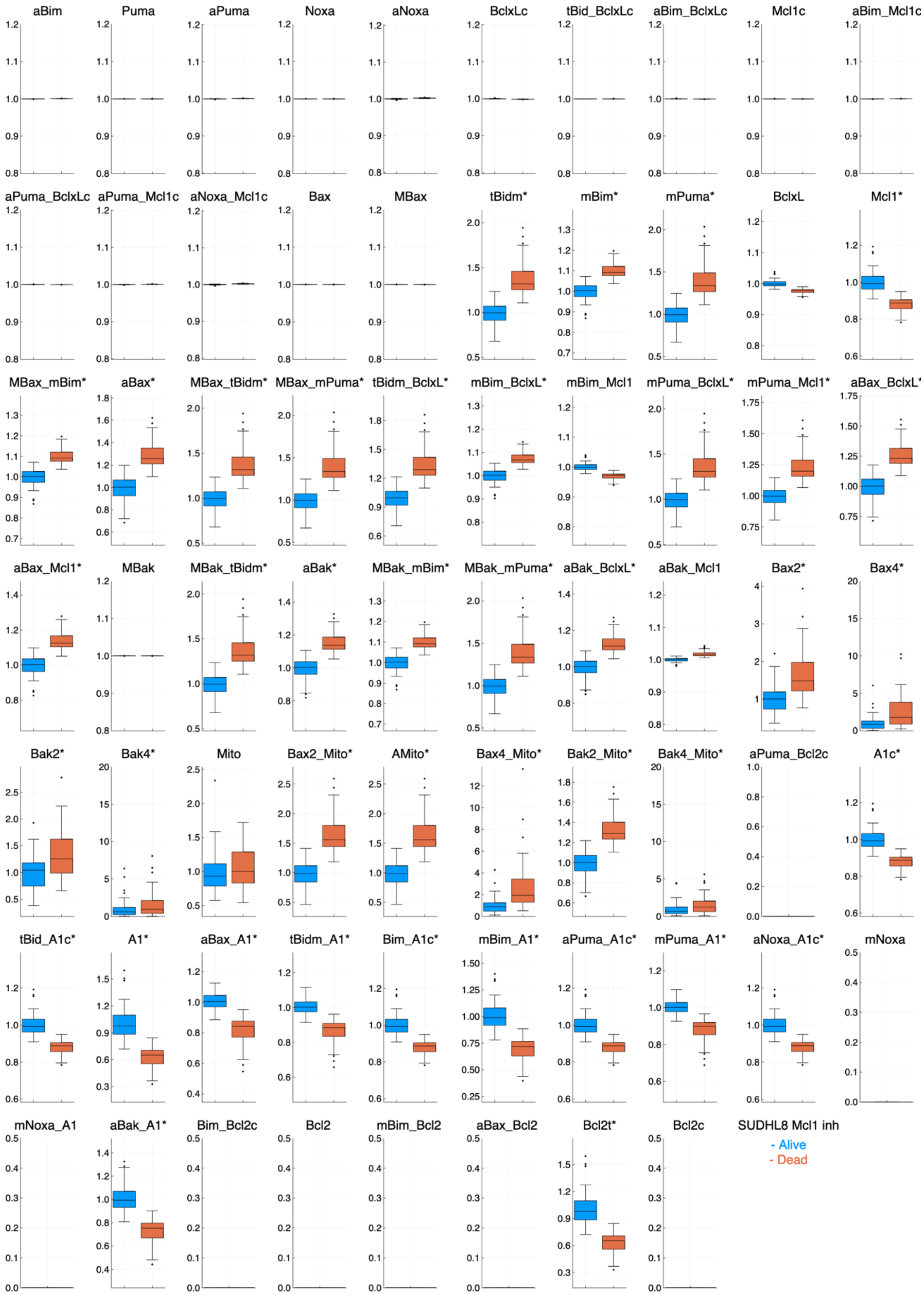

Supplementary Figure 13. Differences in initial Protein abundances in SUDHL8 cells between cells that survive (Blue box) and cells that undergo death (Red box) in response to MCL1 inhibition. * = p<0.05 by two-sample KS test. Median is indicated by a line, with the box extending

to the first and third quartile. Whiskers indicate the range of the data with suspected outliers plotted individually.

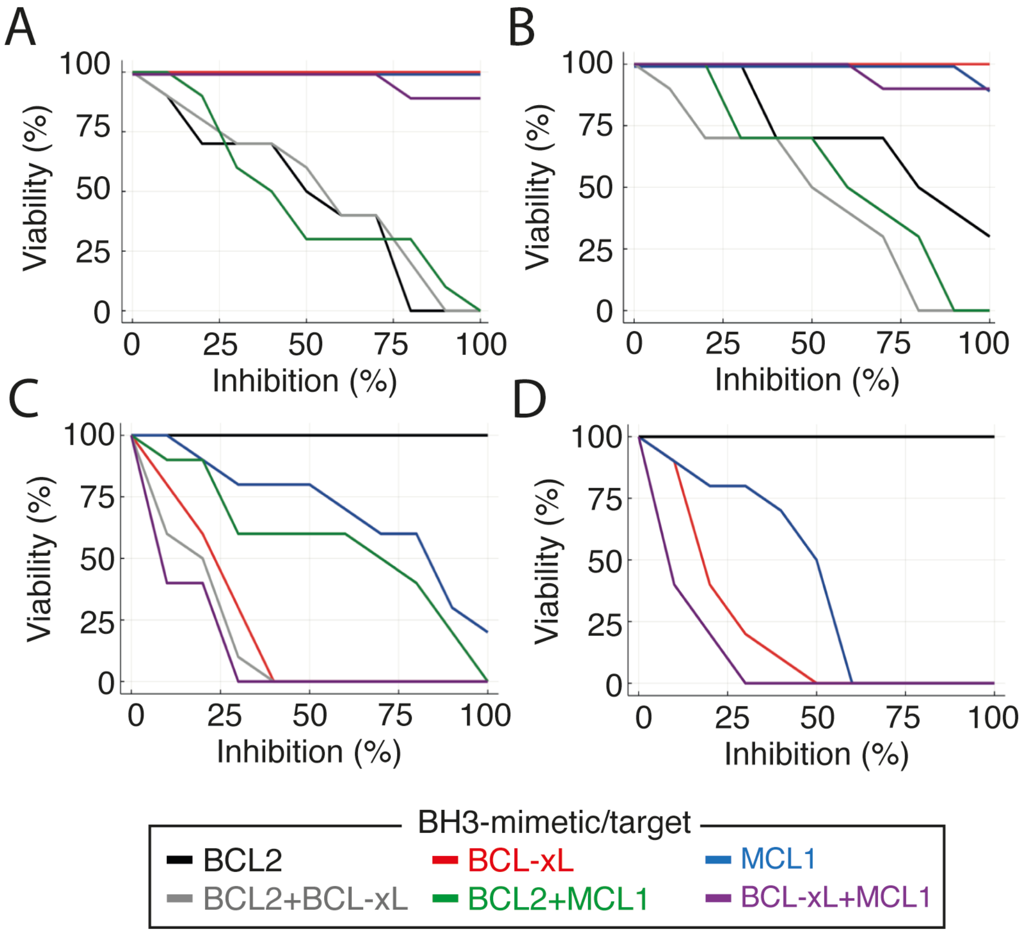

Supplementary Figure 14. (A) Model predicts synergy between BCL2 and MCL1 inhibitors (green curve) at an intermediate range of inhibition in the RIVA cell line but no synergy between BCL2 and BCL-xL inhibitors (grey curve) and between BCL-xL and MCL1 inhibitors (purple curve). (B) In U2932, the model predicts synergy between BCL2 and BCL-xL inhibitors and between BCL2 and MCL1 but not between BCL-xL and MCL1. (C) In RC-K8, model predicts synergy between BCL-xL and MCL1 inhibitors and BCL2 and BCL-xL inhibitors but not between BCL2 and MCL1 inhibitors. (D) In SUDHL8, the model predicts synergy between BCL-xL and MCL1 inhibitors.

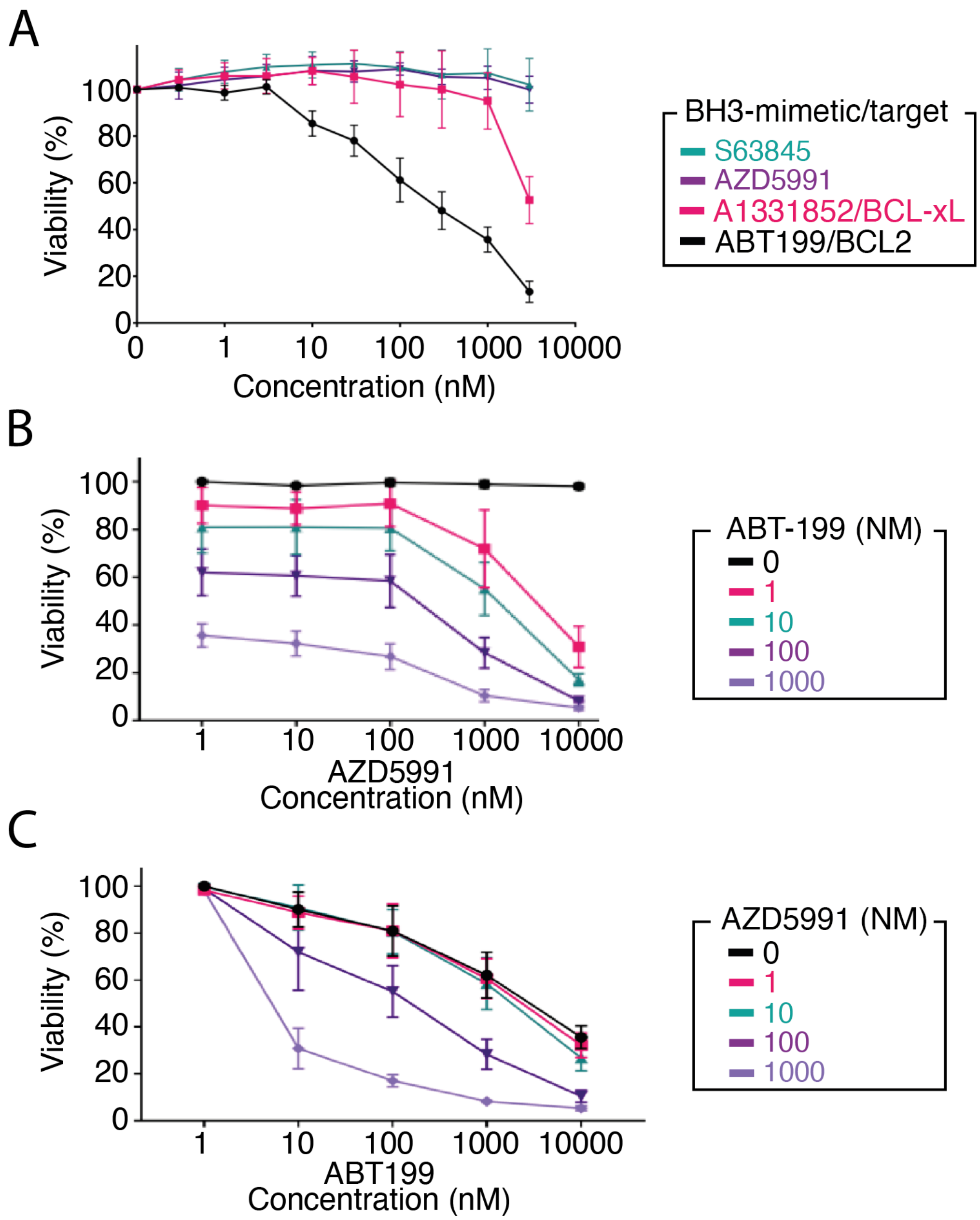

Supplementary Figure 15. (A) Experimental data showing the % viability in response to the BH3-mimetics ABT-199 (Black curve), A1331852 (magenta curve), S63845 (teal curve) and AZD5991 (purple curve) as measured by CellTiter-Glo viability assay. (B,C) Experimental data displaying synergy induced by ABT-199 and AZD5991 combination therapy, combined into a heatmap in Figure 4. N=3, error bars indicate standard deviation.

Supplementary Table 1. Parameters used in model

Parameter	Value (units)	Refs.
BID activation	6*e*−6 ($nM^{-1}.s^{-1}$)	27
BID inactivation	0.06 (s^{-1})	27
Active BID (tBID)	60 (nM)	27
BIM activation	6*e*−6 ($nM^{-1}.s^{-1}$)	27
BIM inactivation	0.06 (s^{-1})	27
Active BIM	60 (nM)	27
PUMA activation	1.07*e*−3 ($nM^{-1}.s^{-1}$)	40
PUMA inactivation	1.41 (s^{-1})	40
Active PUMA	60 (nM)	based on 27
NOXA activation	1.07*e*−3 ($nM^{-1}.s^{-1}$)	40
NOXA inactivation	1.41 (s^{-1})	40
Active NOXA	60 (nM)	based on 27
tBID-BCL-xL complex association rate (cytoplasm)	6*e*−5 ($nM^{-1}.s^{-1}$)	27
tBID-BCL-xL complex dissociation rate (cytoplasm)	0.06 (s^{-1})	27
tBID-BCL-xL complex association rate (MOM)	6*e*−9 ($nM^{-1}.s^{-1}$)	based on 27
tBID-BCL-xL complex dissociation rate (MOM)	0.06 (s^{-1})	based on 27
BIM-BCL-xL complex association rate (cytoplasm)	5.5*e*−5 ($nM^{-1}.s^{-1}$)	3
BIM-BCL-xL complex dissociation rate (cytoplasm)	4.4*e*−4 (s^{-1})	3
BIM-BCL-xL complex association rate (MOM)	5.5*e*−7 ($nM^{-1}.s^{-1}$)	based on 3
BIM-BCL-xL complex dissociation rate (MOM)	4.4*e*−4 (s^{-1})	based on 3
PUMA-BCL-xL complex association rate (cytoplasm)	8.63*e*−5 ($nM^{-1}.s^{-1}$)	3
PUMA-BCL-xL complex dissociation rate (cytoplasm)	4.4*e*−4 (s^{-1})	3
PUMA-BCL-xL complex association rate (MOM)	8.63*e*−7 ($nM^{-1}.s^{-1}$)	based on 3
PUMA-BCL-xL complex dissociation rate (MOM)	4.4*e*−4 (s^{-1})	based on 3
BIM-MCL1 complex association rate (cytoplasm/MOM)	7.8*e*−6 ($nM^{-1}.s^{-1}$)	3
BIM-MCL1 complex dissociation rate (cytoplasm/MOM)	2.17e^{-3} (s^{-1})	3

PUMA-MCL1 complex association rate (cytoplasm)	$1.63e{-4}$ ($nM^{-1}.s^{-1}$)	[3]
PUMA-MCL1 complex dissociation rate (cytoplasm)	$5.21e^{-3}$ (s^{-1})	[3]
PUMA-MCL1 complex association rate (MOM)	$1.63e{-6}$ ($nM^{-1}.s^{-1}$)	based on [3]
PUMA-MCL1 complex dissociation rate (MOM)	$5.21e^{-3}$ (s^{-1})	based on [3]
NOXA-MCL1 complex association rate (cytoplasm)	$1.68e{-5}$ ($nM^{-1}.s^{-1}$)	[3]
NOXA-MCL1 complex dissociation rate (cytoplasm)	$1.39e^{-3}$ (s^{-1})	[3]
BIM-BCL2 complex association rate (cytoplasm)	$4.48e{-5}$ ($nM^{-1}.s^{-1}$)	[3]
BIM-BCL2 complex dissociation rate (cytoplasm)	$1.12e^{-4}$ (s^{-1})	[3]
BIM-BCL2 complex association rate (MOM)	$4.48e{-7}$ ($nM^{-1}.s^{-1}$)	based on [3]
BIM-BCL2 complex dissociation rate (MOM)	$1.12e^{-4}$ (s^{-1})	based on [3]
PUMA-BCL2 complex association rate (cytoplasm)	$4.91e{-5}$ ($nM^{-1}.s^{-1}$)	[3]
PUMA-BCL2 complex dissociation rate (cytoplasm)	$1.57e^{-4}$ (s^{-1})	[3]
PUMA-BCL2 complex association rate (MOM)	$4.91e{-7}$ ($nM^{-1}.s^{-1}$)	based on [3]
PUMA-BCL2 complex dissociation rate (MOM)	$1.57e^{-4}$ (s^{-1})	based on [3]
tBID-A1 complex association rate (cytoplasm)	$8.2e{-4}$ ($nM^{-1}.s^{-1}$)	[41]
tBID-A1 complex dissociation rate (cytoplasm)	$1.55e^{-4}$ (s^{-1})	[41]
tBID-A1 complex association rate (MOM)	$8.2e{-7}$ ($nM^{-1}.s^{-1}$)	[41]
BAX + BAX −→ BAX2	$8.5714e^{-4}$ ($nM^{-1}.s^{-1}$)	[27]
BAX2 −→ BAX + BAX	0.06 (s^{-1})	[27]
tBID-A1 complex dissociation rate (MOM)	$1.55e^{-4}$ (s^{-1})	[41]
BIM-A1 complex association rate (cytoplasm)	$7.8e{-4}$ ($nM^{-1}.s^{-1}$)	based on [41]
BIM-A1 complex dissociation rate (cytoplasm)	$3.95e^{-5}$ (s^{-1})	based on [41]
BIM-A1 complex association rate (MOM)	$7.8e{-6}$ ($nM^{-1}.s^{-1}$)	based on [41]
BIM-A1 complex dissociation rate (MOM)	$3.95e^{-5}$ (s^{-1})	based on [41]
PUMA-A1 complex association rate (cytoplasm)	$5.9e{-3}$ ($nM^{-1}.s^{-1}$)	based on [41]

PUMA-A1 complex dissociation rate (cytoplasm)	$5e{-4}$ (s^{-1})	based on [41]
PUMA-A1 complex association rate (MOM)	$5.9e{-5}$ ($nM^{-1}.s^{-1}$)	based on [41]
PUMA-A1 complex dissociation rate (MOM)	$5e{-4}$ (s^{-1})	based on [41]
NOXA-A1 complex association rate (cytoplasm)	$5.9e{-3}$ ($nM^{-1}.s^{-1}$)	based on [41]
NOXA-A1 complex dissociation rate (cytoplasm)	$1e{-3}$ (s^{-1})	based on [41]
tBID-BAX complex association rate	$6e{-6}$ ($nM^{-1}.s^{-1}$)	[27]
tBID-BAX complex dissociation rate	0.06 (s^{-1})	[27]
tBID activated BAX	60 (nM)	[27]
BIM-BAX complex association rate	$2.07e{-3}$ ($nM^{-1}.s^{-1}$)	based on [3]
BIM-BAX complex dissociation rate	0.06 (s^{-1})	based on [3]
BIM activated BAX	60 (nM)	based on [27]
PUMA-BAX complex association rate	$6e{-6}$ ($nM^{-1}.s^{-1}$)	based on [3]
PUMA-BAX complex dissociation rate	0.06 (s^{-1})	based on [3]
PUMA activated BAX	60 (nM)	based on [27]
tBID-BAK complex association rate	$6e{-6}$ ($nM^{-1}.s^{-1}$)	[27]
tBID-BAK complex dissociation rate	0.06 (s^{-1})	[27]
tBID activated BAK	60 (nM)	[27]
BIM-BAK complex association rate	$2.07e{-3}$ ($nM^{-1}.s^{-1}$)	based on [3]
BIM-BAK complex dissociation rate	0.06 (s^{-1})	based on [3]
BIM activated BAK	60 (nM)	based on [27]
PUMA-BAK complex association rate	$6e{-6}$ ($nM^{-1}.s^{-1}$)	based on [3]
PUMA-BAK complex dissociation rate	0.06 (s^{-1})	based on [3]
PUMA activated BAK	60 (nM)	based on [27]
BCL-xL-BAX complex association rate	$5.18e{-7}$ ($nM^{-1}.s^{-1}$)	[3]
BCL-xL-BAX complex dissociation rate	$4.4e{-4}$ (s^{-1})	[3]
MCL1-BAX complex association rate	$3.25e{-7}$ ($nM^{-1}.s^{-1}$)	[3]
MCL1-BAX complex dissociation rate	$2.6e{-4}$ (s^{-1})	[3]
BCL2-BAX complex association rate	$3.24e{-5}$ ($nM^{-1}.s^{-1}$)	[3]
BCL2-BAX complex dissociation rate	$3.36e^{-4}$ (s^{-1})	[3]
A1-BAX complex association rate	$1.4e{-6}$ ($nM^{-1}.s^{-1}$)	[41]
A1-BAX complex dissociation rate	$3.36e^{-5}$ (s^{-1})	[41]
BCL-xL-BAK complex association rate	$5.5e{-6}$ ($nM^{-1}.s^{-1}$)	[3]
BCL-xL-BAK complex dissociation rate	$4.4e{-4}$ (s^{-1})	[3]
MCL1-BAK complex association rate	$3.25e{-7}$ ($nM^{-1}.s^{-1}$)	[3]
MCL1-BAK complex dissociation rate	$2.6e{-4}$ (s^{-1})	[3]
A1-BAK complex association rate	$6.03e{-7}$ ($nM^{-1}.s^{-1}$)	[41]
A1-BAK complex dissociation rate	$2.6e{-4}$ (s^{-1})	[41]
BAX2 + BAX2 −→ BAX4	$8.5714e^{-4}$ ($nM^{-1}.s^{-1}$)	[27]
BAX4 −→ BAX2 + BAX2	0.06 (s^{-1})	[27]

BAK + BAK −→ BAK2	$8.5714e^{-4}$ ($nM^{-1}.s^{-1}$)	[27]
BAK2 −→ BAK + BAK	0.06 (s^{-1})	[27]
BAK2 + BAK2 −→ BAK4	$8.5714e^{-4}$ ($nM^{-1}.s^{-1}$)	[27]
BAK4 −→ BAK2 + BAK2	0.06 (s^{-1})	[27]
BAX2-MITO association	8.5714 ($nM^{-1}.s^{-1}$)	[27]
BAX2-MITO dissociation	0.06 (s^{-1})	[27]
BAX2-induced MOMP	60 (nM)	[27]
BAX4-MITO association	8.5714 ($nM^{-1}.s^{-1}$)	[27]
BAX4-MITO dissociation	0.06 (s^{-1})	[27]
BAX4-induced MOMP	60 (nM)	[27]
BAK2-MITO association	8.5714 ($nM^{-1}.s^{-1}$)	[27]
BAK2-MITO dissociation	0.06 (s^{-1})	[27]
BAK2-induced MOMP	60 (nM)	[27]
BAK4-MITO association	8.5714 ($nM^{-1}.s^{-1}$)	[27]
BAK4-MITO dissociation	0.06 (s^{-1})	[27]
BAK4-induced MOMP	60 (nM)	[27]
MOMP −→ MITO	$1.155245e^{-1}$ (s^{-1})	[27]
BAX −→ MOM	$1.16e^{-2}$ (s^{-1})	[3]
BAX −→ cytoplasm	$1.16e^{-1}$ (s^{-1})	[3]
tBID −→ MOM	$1.16e^{-2}$ (s^{-1})	based on [3]
tBID −→ cytoplasm	$1.16e^{-3}$ (s^{-1})	[3]
BIM −→ MOM	$1.16e^{-2}$ (s^{-1})	[3]
BIM −→ cytoplasm	$1.16e^{-3}$ (s^{-1})	[3]
PUMA −→ MOM	$1.16e^{-2}$ (s^{-1})	[3]
PUMA −→ cytoplasm	$1.16e^{-3}$ (s^{-1})	[3]
BCL-xL −→ MOM	$1.155245e^{-2}$ (s^{-1})	based on [27]
BCL-xL −→ cytoplasm	$1.155245e^{-3}$ (s^{-1})	based on [27]
MCL1 −→ MOM	$1.155245e^{-2}$ (s^{-1})	based on [27]
MCL1 −→ cytoplasm	$1.155245e^{-3}$ (s^{-1})	based on [27]
BCL2 −→ MOM	$1.155245e^{-2}$ (s^{-1})	[27]
BCL2 −→ cytoplasm	$1.155245e^{-3}$ (s^{-1})	[27]
A1 −→ MOM	$1.155245e^{-2}$ (s^{-1})	based on [27]
A1 −→ cytoplasm	$1.155245e^{-3}$ (s^{-1})	based on [27]
Apoptotic signal expression	$6.11505e^{-2}$ ($nM.s^{-1}$)	[27]
Apoptotic signal degradation	$1.44406e^{-3}$ (s^{-1})	[27]
BID expression	*cell line-specific*	this work
BID degradation	$1.155245e^{-2}$ (s^{-1})	[27]
BIM expression	*cell line-specific*	this work
BIM degradation	$6.91e^{1}$ (s^{-1})	[3]
PUMA expression	*cell line-specific*	this work
PUMA degradation	$6.8e^{-1}$ (s^{-1})	[3]

NOXA expression	*cell line-specific*	this work
NOXA degradation	9.36e^{-1} (s^{-1})	this work
BCL-xL expression	*cell line-specific*	this work
BCL-xL degradation	1.155245e^{-2} (s^{-1})	[27]
MCL1 expression	*cell line-specific*	this work
MCL1 degradation	1.155245e^{-2} (s^{-1})	[27]
BCL2 expression	*cell line-specific*	this work
BCL2 degradation	6.912e^{-1} (s^{-1})	[27]
A1 expression	*cell line-specific*	this work
A1 degradation	9.36e^{-1} (s^{-1})	this work
BAX expression	*cell line-specific*	this work
BAX degradation	1.155245e^{-2} (s^{-1})	[27]
BAK expression	*cell line-specific*	this work
BAK degradation	6.82e^{-2} (s^{-1})	[27]
tBID-BCL-xL degradation (cytoplasm/MOM)	1.155245e^{-2} (s^{-1})	based on [27]
BIM-BCL-xL degradation (cytoplasm/MOM)	9.93e^{-1}/9.75e^{-1} (s^{-1})	[3]
PUMA-BCL-xL degradation (cytoplasm/MOM)	6.75e^{-2} (s^{-1})	[3]
BIM-MCL1 degradation (cytoplasm/MOM)	9.93e^{-1} (s^{-1})	[3]
PUMA-MCL1 degradation (cytoplasm/MOM)	6.93e^{-2} (s^{-1})	[3]
NOXA-MCL1 degradation	3.75e^{-2} (s^{-1})	based on [3]
BIM-BCL2 degradation (cytoplasm/MOM)	6.93e^{-1}/6.75e^{-1} (s^{-1})	[3]
PUMA-BCL2 degradation (cytoplasm/MOM)	6.75e^{-2} (s^{-1})	[3]
tBID-A1 degradation (cytoplasm/MOM)	6.75e^{-2} (s^{-1})	based on [41]
BIM-A1 degradation (cytoplasm/MOM)	6.93e^{-1}/ (s^{-1})	[41]
PUMA-A1 degradation (cytoplasm/MOM)	6.93e^{-2} (s^{-1})	based on [41]
NOXA-MCL1 degradation	6.75e^{-2} (s^{-1})	based on [3]
tBID-BAX degradation	6.75e^{-2} (s^{-1})	[27]
BIM-BAX degradation	6.93e^{-2} (s^{-1})	[3]
PUMA-BAX degradation	6.93e^{-2} (s^{-1})	[3]
tBID-BAK degradation	6.75e^{-2} (s^{-1})	[27]
BIM-BAK degradation	6.93e^{-2} (s^{-1})	[3]
PUMA-BAK degradation	6.93e^{-2} (s^{-1})	[3]
BAX-BCL-xL degradation	1.155245e^{-2} (s^{-1})	[3]
BAX-MCL1 degradation	6.93e^{-2} (s^{-1})	[3]
BAX-BCL2 degradation	6.93e^{-2} (s^{-1})	[27]
BAX-A1 degradation	1.155245e^{-2} (s^{-1})	[41]
BAK-BCL-xL degradation	6.93e^{-2} (s^{-1})	[3]
BAK-MCL1 degradation	6.75e^{-2} (s^{-1})	[3]
BAK-A1 degradation	6.75e^{-2} (s^{-1})	[41]
BID degradation (cytoplasm/MOM)	1.155245e^{-2} (s^{-1})	[27]
BIM degradation (cytoplasm/MOM)	6.91e^{-1} (s^{-1})	[3]

PUMA degradation (cytoplasm/MOM)	$6.8e^{-1}$ (s^{-1})	[3]

Supplementary Table 2. Cell line-specific parameters used in model

Cell line-specific parameters	RC-K8	RIVA	SU-DHL-8	SU-DHL-10	U-2932	U-2946
BID expression	427.0945	1.7709	577.0945	17.7094	1.7709	17.7094
BIM expression	$7.05e^{3}$	$1.78e^{4}$	$7.02e^{4}$	$2.5e^{3}$	$1.77e^{4}$	$1.21e^{4}$
PUMA expression	$6.44e^{4}$	$1.23e^{4}$	$9.36e^{3}$		$2.36e^{3}$	
NOXA expression	35581.818	22112.1818	230542.182	19818.18	55818.18	14081.818
BCL-xL expression	6076.0454	65.0454	3860.4548		2.46	
MCL1 expression	30374.8	17.48	40818.748	3874.8	$8.37e^{3}$	3874.8
BCL2 expression	42.6407	4264.07			$2.13e^{4}$	
BCL2A1 expression	17.35		117.35			
BAX expression	8856.4059	3001.6406	5941.6406	151.6406	12016.406	701.6406
BAK expression	$7.94e^{4}$	$1.3e^{3}$	$9.91e^{4}$	$1.08e^{4}$	$9.5e^{2}$	8.75e3
BAX-BCL2 degradation	0.0693	0.0693			**0.1693**	
BAK-BCL-xL degradation	**0.1493**	0.0693	0.0693		0.0693	
BAX-BCL-xL degradation	**0.004**	0.0116	0.0116		0.0116	
NOXA-MCL1 degradation	0.0375	0.0375	0.0375	**5.98**	**4.98**	**3.78**
BAK-MCL1 degradation	0.0675	0.0675	0.0675	**0.1375**	0.0675	0.0675
aBIM-MCL1c degradation	0.993	0.993	0.993	**0.193**	0.993	**0.0193**
mBIM-MCL1 degradation	0.993	0.993	0.993	**0.093**	0.993	**0.0093**

Supplementary References

1. Loriaux PM, Tesler G, Hoffmann A. Characterizing the relationship between steady state and response using analytical expressions for the steady states of mass action models. PLoS computational biology. 2013;9(2):e1002901-e1002901.
2. Kim M-S, Pinto SM, Getnet D, et al. A draft map of the human proteome. Nature. 2014;509(7502):575–581-575–581.
3. Song T, Wang P, Yu X, et al. Systems analysis of phosphorylation-regulated Bcl-2 interactions establishes a model to reconcile the controversy over the significance of Bcl-2 phosphorylation. British journal of pharmacology. 2019;176(3):491–504-491–504.
4. Smith VM, Dietz A, Henz K, et al. Specific interactions of BCL-2 family proteins mediate sensitivity to BH3-mimetics in diffuse large B-cell lymphoma. Haematologica. 2020;105(8):2150.
5. Abràmoff MD, Magalhães PJ, Ram SJ. Image processing with ImageJ. Biophotonics international. 2004;11(7):36–42-36–42.
6. Letai A, Bassik MC, Walensky LD, Sorcinelli MD, Weiler S, Korsmeyer SJ. Distinct BH3 domains either sensitize or activate mitochondrial apoptosis, serving as prototype cancer therapeutics. Cancer cell. 2002;2(3):183–192-183–192.
7. Chen L, Willis SN, Wei A, et al. Differential targeting of prosurvival Bcl-2 proteins by their BH3-only ligands allows complementary apoptotic function. Molecular cell. 2005;17(3):393–403-393–403.
8. Certo M, Moore VDG, Nishino M, et al. Mitochondria primed by death signals determine cellular addiction to antiapoptotic BCL-2 family members. Cancer cell. 2006;9(5):351–365-351–365.
9. Billen LP, Kokoski CL, Lovell JF, Leber B, Andrews DW. Bcl-XL inhibits membrane permeabilization by competing with Bax. PLoS biology. 2008;6(6):e147-e147.
10. Chipuk JE, Green DR. How do BCL-2 proteins induce mitochondrial outer membrane permeabilization? Trends in cell biology. 2008;18(4):157–164-157–164.
11. Cui J, Chen C, Lu H, Sun T, Shen P. Two independent positive feedbacks and bistability in the Bcl-2 apoptotic switch. PloS one. 2008;3(1):e1469-e1469.
12. Lovell JF, Billen LP, Bindner S, et al. Membrane binding by tBid initiates an ordered series of events culminating in membrane permeabilization by Bax. Cell. 2008;135(6):1074–1084-1074–1084.
13. Leber B, Lin J, Andrews DW. Still embedded together binding to membranes regulates Bcl-2 protein interactions. Oncogene. 2010;29(38):5221–5230-5221–5230.
14. Llambi F, Moldoveanu T, Tait SWG, et al. A unified model of mammalian BCL-2 protein family interactions at the mitochondria. Molecular cell. 2011;44(4):517–531-517–531.
15. Aranovich A, Liu Q, Collins T, et al. Differences in the mechanisms of proapoptotic BH3 proteins binding to Bcl-XL and Bcl-2 quantified in live MCF-7 cells. Molecular cell. 2012;45(6):754–763-754–763.
16. Hata AN, Engelman JA, Faber AC. The BCL2 family: key mediators of the apoptotic response to targeted anticancer therapeutics. Cancer discovery. 2015;5(5):475–487-475–487.
17. Shamas-Din A, Kale J, Leber B, Andrews DW. Mechanisms of action of Bcl-2 family proteins. Cold Spring Harbor perspectives in biology. 2013;5(4):a008714-a008714.

18. Fussenegger M, Bailey JE, Varner J. A mathematical model of caspase function in apoptosis. Nature biotechnology. 2000;18(7):768–774-768–774.
19. Bentele M, Lavrik I, Ulrich M, et al. Mathematical modeling reveals threshold mechanism in CD95-induced apoptosis. The Journal of cell biology. 2004;166(6):839–851-839–851.
20. Eissing T, Conzelmann H, Gilles ED, Allgower F, Bullinger E, Scheurich P. Bistability analyses of a caspase activation model for receptor-induced apoptosis. Journal of Biological Chemistry. 2004;279(35):36892–36897-36892–36897.
21. Stucki JW, Simon H-U. Mathematical modeling of the regulation of caspase-3 activation and degradation. Journal of theoretical biology. 2005;234(1):123–131-123–131.
22. Bagci EZ, Vodovotz Y, Billiar TR, Ermentrout GB, Bahar I. Bistability in apoptosis: roles of bax, bcl-2, and mitochondrial permeability transition pores. Biophysical journal. 2006;90(5):1546–1559-1546–1559.
23. Legewie S, Blüthgen N, Herzel H. Mathematical modeling identifies inhibitors of apoptosis as mediators of positive feedback and bistability. PLoS computational biology. 2006;2(9):e120-e120.
24. Rehm M, Huber HJ, Dussmann H, Prehn JHM. Systems analysis of effector caspase activation and its control by X-linked inhibitor of apoptosis protein. The EMBO journal. 2006;25(18):4338–4349-4338–4349.
25. Chen C, Cui J, Lu H, Wang R, Zhang S, Shen P. Modeling of the role of a Bax-activation switch in the mitochondrial apoptosis decision. Biophysical Journal. 2007;92(12):4304–4315-4304–4315.
26. Chen C, Cui J, Zhang W, Shen P. Robustness analysis identifies the plausible model of the Bcl-2 apoptotic switch. FEBS letters. 2007;581(26):5143–5150-5143–5150.
27. Albeck JG, Burke JM, Spencer SL, Lauffenburger DA, Sorger PK. Modeling a snap-action, variable-delay switch controlling extrinsic cell death. PLoS biology. 2008;6(12):e299-e299.
28. Lindner AU, Concannon CnG, Boukes GJ, et al. Systems analysis of BCL2 protein family interactions establishes a model to predict responses to chemotherapy. Cancer research. 2013;73(2):519–528-519–528.
29. Würstle ML, Zink E, Prehn JHM, Rehm M. From computational modelling of the intrinsic apoptosis pathway to a systems-based analysis of chemotherapy resistance: achievements, perspectives and challenges in systems medicine. Cell death & disease. 2014;5(5):e1258–e1258-e1258–e1258.
30. Zhao L, Sun T, Pei J, Ouyang Q. Mutation-induced protein interaction kinetics changes affect apoptotic network dynamic properties and facilitate oncogenesis. Proceedings of the National Academy of Sciences. 2015;112(30):E4046–E4054-E4046–E4054.
31. Bleicken S, Hantusch A, Das KK, Frickey T, Garcia-Saez AJ. Quantitative interactome of a membrane Bcl-2 network identifies a hierarchy of complexes for apoptosis regulation. Nature communications. 2017;8(1):1–15-11–15.
32. Kale J, Osterlund EJ, Andrews DW. BCL-2 family proteins: changing partners in the dance towards death. Cell Death & Differentiation. 2018;25(1):65–80-65–80.
33. Kim H, Rafiuddin-Shah M, Tu H-C, et al. Hierarchical regulation of mitochondrion-dependent apoptosis by BCL-2 subfamilies. Nature cell biology. 2006;8(12):1348–1358-1348–1358.

34. Chen H-C, Kanai M, Inoue-Yamauchi A, et al. An interconnected hierarchical model of cell death regulation by the BCL-2 family. Nature cell biology. 2015;17(10):1270–1281-1270–1281.
35. Campbell KJ, Tait SWG. Targeting BCL-2 regulated apoptosis in cancer. Open biology. 2018;8(5):180002-180002.
36. Xiang W, Yang C-Y, Bai L. MCL-1 inhibition in cancer treatment. OncoTargets and therapy. 2018;11(7301-7301.
37. Willis SN, Fletcher JI, Kaufmann T, et al. Apoptosis initiated when BH3 ligands engage multiple Bcl-2 homologs, not Bax or Bak. Science. 2007;315(5813):856–859-856–859.
38. Rehm M, Dussmann H, Janicke RU, Tavare JM, Kogel D, Prehn JHM. Single-cell fluorescence resonance energy transfer analysis demonstrates that caspase activation during apoptosis is a rapid process: role of caspase-3. Journal of Biological Chemistry. 2002;277(27):24506–24514-24506–24514.
39. Tyas L, Brophy VA, Pope A, Rivett AJ, Tavaré JM. Rapid caspase-3 activation during apoptosis revealed using fluorescence-resonance energy transfer. EMBO reports. 2000;1(3):266–270-266–270.
40. Liu B, Bhatt D, Oltvai ZN, Greenberger JS, Bahar I. Significance of p53 dynamics in regulating apoptosis in response to ionizing radiation and polypharmacological strategies. Scientific Reports. 2014;4(1):1–12-11–12.
41. Mathieu A-L, Sperandio O, Pottiez V, et al. Identification of small inhibitory molecules targeting the Bfl-1 anti-apoptotic protein that alleviates resistance to ABT-737. Journal of Biomolecular Screening. 2014;19(7):1035–1046-1035–1046.
